# Emerging horizons: A Rainbow Model for the sustainable implementation of Rain Classroom in vocational nursing education

**DOI:** 10.1371/journal.pone.0331848

**Published:** 2025-09-29

**Authors:** Lina Wang, Peng Li, Jie Li, Mengqi Chen, Shuang Xi Guo, Youcong Zhu, Xiaolei Gao, Cuiping Li, Anna Ma

**Affiliations:** 1 School of Nursing, Henan Medical University, Xinxiang, China; 2 Xinzhou Modern Healthcare and Nursing Vocational College, Xinzhou, China; 3 The First Affiliated Hospital of Henan Medical University, Xinxiang, China; 4 Yiyang Medical College, Hunan, China; 5 School of Basic Medical Sciences, Henan Medical University, Xinxiang, China; University of Luzon, PHILIPPINES

## Abstract

**Background:**

The COVID-19 pandemic has accelerated several social changes, particularly the shift in education towards online environments. Digital technologies are being extensively used in educational practices, but current research mostly focuses on describing digital teaching practices, with few studies summarising practical experiences and extracting universal principles and patterns.

**Objective:**

To summarise the practical experience of using Rain Classroom in information technology teaching tools, and to extract universal principles and patterns.

**Method:**

From July 1 to December 1 2023, eight semi-structured qualitative interviews were conducted with teachers in Hunan, China, who used Rain Classroom for digital teaching. Through multi-level coding, key themes were progressively distilled from interview data, combined with cross-level mapping from the multidimensional perspectives of the Rainbow Framework, ultimately abstracting theoretical principles.

**Results:**

The convenience, interactivity and diversified learning functions of Rain Classroom’s digital teaching are highlighted in this study. Through the summarisation of practical experience, a theoretical model was established, focusing on Resilience, Adaptability, Inclusivity, Nurturing Continuous Improvement, Balanced Evaluations, Optimal User Experience and Weathering Challenges with Comprehensive Enablers.

## Introduction

The COVID-19 pandemic has accelerated the digitisation of teaching [1]. With the advent of the “Internet+” era, the shortcomings of traditional teaching methods, such as limited educational resources and outdated information, have become increasingly prominent, positing new requirements for educational reform and innovation. Currently, countries around the world are vigorously developing information technology, which in turn is driving the transformation of new types of information-based and digitised education. Many countries have joined the ranks of educational informatisation reform. For example, China has explicitly stated in the “Ten-Year Plan for the Development of Educational Informatisation (2011-2020)” that it aims to promote the deep integration of information technology into education, thereby comprehensively improving the quality of education [2].

Blended learning is an educational model that organically combines online learning (using digital resources and tools) with face-to-face classroom teaching. It enables online and offline learning experiences to complement each other through well-designed teaching and learning activities in order to optimise the learning process and improve outcomes [3]. The core feature lies in the use of learning data analytics to dynamically understand the individual needs of students and to provide adapted personalised learning paths and continuous support. This learning approach has been widely adopted globally, especially in the process of education informatisation reform, which makes learning more flexible and personalised by breaking down traditional teaching time and location constraints [4].

Rain Classroom is a digital learning tool based on the WeChat platform and is widely used in the field of education in China [5]. As a digital learning tool, Rain Classroom provides a wide range of teaching functions and tools aimed at improving teaching efficiency and student engagement. Through seamless integration with WeChat, Rain Classroom allows teachers and students to use their WeChat accounts for online interaction and learning activities [6].

Rain Classroom, as a digital teaching tool, has been referred to as the “new symbol of the development of university teaching informatisation in China” [7]. It can be used on mobile WeChat, on a computer webpage or as a standalone application, greatly facilitating teachers’ teaching and students’ learning [8]. Rain Classroom has a rich set of digital teaching functions, including Real-time Interaction, Multimedia Support, Assignments and Quizzes, Course Management, Collaborative Learning, Data Analysis and Reporting, and Mobile Learning. Since its launch in 2016, it has been implemented and applied in teaching at many universities. By April 2023, the global user base had reached 90 million.

As a digital teaching tool, Rain Classroom has evolved to version 6.0, comprehensively covering functions in the pre-class, in-class and post-class stages, effectively promoting the development of digital teaching [9]. However, current research on Rain Classroom mainly focuses on the construction of different course teaching models and the evaluation of teaching effectiveness [10]. In order to further promote the healthy development and internationalisation of this digital teaching tool, it is necessary to examine the theory of the teaching model on which it is based. Research in this area is still lacking; therefore, this paper takes Rain Classroom as the research object, objectively describes its teaching model, analyses its core features, enriches its theoretical framework and provides necessary conditions for the further development of digital teaching in schools.

The Rainbow Theory Model is a social ecological model first proposed by American scholars in 1993 [11]. This model aims to assist researchers and practitioners in understanding and addressing complex public health and social issues. It provides a comprehensive perspective on problems by organising various potential determinants and intervention policies into different levels. The Rainbow Theory Model emphasises multi-level factors and advocates for integrated interventions to promote changes into healthy behaviours.

The Rainbow Theory Model highlights the interactions between these different levels and argues that effective interventions need to consider the impact of all these levels. This multi-level approach helps identify and address the root causes of unhealthy behaviours, thereby formulating more comprehensive and effective strategies to improve the level of public health [12].

In public health and social science research, the Rainbow Theory Model is widely applied in disease prevention, promotion of healthy behaviours, assessment of intervention effectiveness, as well as other areas. It not only provides an analytical framework for researchers but also offers a theoretical basis for policymakers to implement intervention programmes [13].

The Rainbow Framework is a multi-level dynamic integration theoretical model for digital teaching and learning, based on the socio-ecological structure of the Rainbow Theory Model and incorporating the core practical elements of the Rain Classroom, aiming to systematically analyse, optimise and enhance the adaptability of information-based teaching tools, inclusiveness and sustainability. Through the multi-level interaction mechanism of Rainbow Theory, the framework builds a cross-level collaborative evolution path, from micro-technology operation to macro-teaching design, providing a systematic theoretical tool to help educators realise the theoretical development and international application of digital teaching tools, and to promote the paradigm shift of teaching practice to a deeper level.

This article summarises the practical experience of using Rain Classroom by interviewing and surveying its users. Through multiple case studies, it extracts and summarises the core elements of digital learning in Rain Classroom. Based on this, it has preliminarily constructed a Rainbow Theory Model with the following cores: Weathering Challenges with Comprehensive Enablers, Optimal User Experience, Balanced Teacher and Student Evaluations, Nurturing Continuous Improvement, Inclusive Mobile-Centric Learning, Adaptive Device Accessibility, and Resilient Consistency in Usage. This model aims to help teachers and students better utilise Rain Classroom for informatised teaching.

## Methods

Rain Classroom is a teaching tool that was developed by Tsinghua University and XuetangX that integrates with the popular messaging app WeChat. Currently, research on Rain Classroom mainly focuses on describing teaching practices. In order to further promote the development of digital teaching with Rain Classroom, this study focused on constructing a sustainable development model for blended learning using Rain Classroom. Through case studies and interviews with teachers using Rain Classroom for teaching from July 1 to December 1, 2023, this article will summarise practical experience, extract universal patterns and further advance the theoretical development of digital learning. Firstly, practical experience will be summarised to reveal the core elements of digital learning with Rain Classroom. By analysing the teaching strategies adopted by teachers in different situations and the characteristics of the learning environment, important factors affecting the effectiveness of blended learning will be identified. Secondly, practical experience will be summarised to establish a theoretical model. By comparing and analysing multiple actual cases, common patterns and rules will be revealed and abstracted into theoretical models and frameworks. These models and frameworks can provide guidance for teachers, helping them better apply digital teaching in practice, and provide research directions and theoretical support for educational researchers.

This study was reviewed and approved by the Ethics Committee of Yiyang Medical College (Ethics Number: 2023001), with all participants providing written informed consent prior to interviews and explicitly agreeing to the anonymisation and exclusive academic use of data. The study excluded minors from participation.

### Data collection

In this study, data collection was conducted according to the guidelines and principles of data collection proposed by Yin [14]. Semi-structured interviews were conducted with eight teachers from four different universities in Hunan Province, China. These semi-structured interviews were open-ended, allowing space for participants to discuss topics and express views, while also using a thematic guide to address key issues [15]. Before collecting data, a detailed interview outline was developed based on the research objectives. The interviews mainly covered the background and development history of Rain Classroom teaching, specific methods, courses using Rain Classroom teaching, effectiveness and student satisfaction. When focusing on a specific school, the interview outline was enriched and customised according to the specific situation of the school.

Building on the above work, semi-structured interviews were the main method of collecting data, with each interview lasting 0.5 to 1 hour. The interviewees were teachers using Rain Classroom for teaching, with two teachers from each school, totalling eight teachers. All participants were contacted in advance and agreed to participate in the study. Before each interview began, the interviewee again confirmed their agreement to participate and then signed the informed consent form. Prior to the formal interviews, a pilot interview was conducted with one teacher and evaluated for its effectiveness. All interviews were conducted in Chinese by the first author and recorded, then translated into English.

### Analysis

This study drew on the advice of Yin [16] who designed the following case study process: first, using Eisenhardt’s approach [17], each case was studied individually in a single case analysis. Based on the results of these individual case studies, a cross-case analysis was conducted to compare the results across cases. Transcripts of interviews were then coded using thematic content analysis. Themes extracted from the interviews were incorporated into the thematic analysis along with themes that emerged during the interviews. The thematic coding structure thus formed was evaluated by four assessors. Next, these assessors independently coded the interview data. The aim of this approach was to enhance reliability among assessors by selecting assessors from different backgrounds and to increase inter-subject judgment across different disciplines. After comparing the independent coding assessments, they were combined to construct the final coding structure.

## Results

### The extent of teachers’ use of Rain Classroom

The interviews revealed that teachers of all age groups had been using Rain Classroom on computers at least once a week in their teaching for a period of over two years. The average usage of “Rain Classroom” was twice a week (Table 1). This indicates that Rain Classroom is widely used among teachers in Hunan Vocational Nursing Schools. In terms of functionality, the teachers were using a wide range of functions provided by Rain Classroom, including online teaching, real-time interaction and discussion, simulation exercises and case analysis, assignment posting and grading, online quizzes and assessments, course evaluation and feedback, personalised guidance, operational training and pre-learning effect testing; in other words, almost all functions provided by Rain Classroom. The widespread and in-depth application of Rain Classroom among teachers in Hunan Vocational Nursing Schools not only indicates its wide acceptance but also demonstrates its capacity to meet the diverse needs of educators as well as its ability to improve the quality of education through modern technology-driven teaching methods.

**Table 1:** Summary of findings and cross-case analysis of the Rain Classroom utilization.

Case	Findings	Similarities	Differences	Patterns/Themes
**School A**	Both teachers have been using Rain Classroom for 2–3 years, conducting more than one session per week. They utilize computers for teaching, focusing on the subjects of Basic Nursing and Health Assessment.	Both students and teachers used the rain classroom for more than a year, and they study at least once a week	Students used more mobile phones to study, and teachers are more inclined to choose computers for rain classroom	Consistent usage patterns Device Preference Discrepancy Mobile-Centric Student Learning Teacher Emphasis on Desktop Efficiency
**School B**	Both teachers have been using Rain Classroom for 2–3 years, conducting sessions weekly. They utilize computers for teaching.	Both students and teachers use the rain class for more than a year, and they study at least once a week		
**School C**	Using Rain Classroom for 2 and 3 years, with a frequency of per week. Both teachers utilize computers for teaching.	Both students and teachers use the rain class for more than a year, and they study at least once a week		
**School D**	Using Rain Classroom for 3 years, with a frequency of per week. Both teachers utilize computers for teaching.	Both students and teachers use the rain class for more than a year, and they study at least once a week		

### Teacher evaluation of Rain Classroom based on functionality

Through the interviews, it was revealed that the main functions the eight teachers mainly used in Rain Classroom included online teaching, real-time interaction and discussion, simulation exercises and case analysis, assignment posting and grading, online quizzes and assessments, resource sharing, personalised guidance, course evaluation and feedback, operational training and pre-learning effect testing.

When asked about their favourite Rain Classroom features, three teachers mentioned that they found the online discussion and interaction features particularly beneficial. One teacher replied, “My favorite feature is online discussion and interaction. This feature allows me to communicate directly with students, answer questions, promote exchange of ideas, and make the course more interactive and engaging.” Another teacher mentioned, “My favorite feature is personalised guidance. Senior teachers can use Rain Classroom to grant access to surgical videos for students, allowing them to watch the videos in advance and ask questions, to which the tutor can provide explanations. Furthermore, the tutor can select a student to perform live surgery (based on typical clinical cases) while other nursing students offer comments and corrections, pointing out deficiencies in the surgical process. The guiding teacher assesses, and then the student performs the entire operation again, with the guiding teacher providing detailed explanations at each step and possibly grading along the way.”

The course evaluation and feedback function was also highly favoured by the teachers. They believed that through Rain Classroom’s digital evaluation function, they could appreciate students’ satisfaction and understanding of the course, and accordingly adjust teaching methods and content to improve the quality of teaching.

### Teacher evaluation of Rain Classroom based on teaching satisfaction

In interviews assessing the teachers’ satisfaction with the use of Rain Classroom, all eight teachers expressed being “extremely satisfied.” This indicates that using Rain Classroom has been beneficial for the teachers. One teacher said, “I am very satisfied with using Rain Classroom for teaching. It provides me with rich tools and resources, making the classroom livelier and more interesting.” Another teacher said, “I am very satisfied with using Rain Classroom for teaching. It allows students to attend classes according to their own schedules, providing greater flexibility.” Additionally, one teacher mentioned, “I am very satisfied with using Rain Classroom for teaching. It provides online interactive features that allow real-time communication and discussion between students and teachers, promoting interactive learning.” Another user stated, “I am very satisfied with using Rain Classroom for teaching. It adds a lot of colour to my course, making teaching more dynamic.”

Based on the results of this interview summarising teachers’ satisfaction with the use of Rain Classroom, we can see that teachers appear to be extremely satisfied with it. This is because it provides a wide range of functions that effectively integrate the theory and practice of nursing education.

### Evaluation of teachers’ opinions on the teaching effectiveness of Rain Classroom

In interviews evaluating teaching effectiveness, all eight teachers believed that Rain Classroom had a positive impact on students’ learning outcomes and achievements (Table 2). For example, one teacher responded, “Students’ performance and results in Rain Classroom are inspiring. They actively participate in the course through discussions and completing assignments, demonstrating a high level of learning motivation.” Additionally, teachers noted that students’ attitudes towards learning were generally positive in the Rain Classroom environment. One teacher said, “In the teaching environment of Rain Classroom, the classroom atmosphere is usually vibrant and interactive. Students actively participate in the course through real-time interaction and discussion in the virtual space, demonstrating good learning states, high levels of concentration and curiosity.” Another teacher mentioned that Rain Classroom fosters a positive interactive classroom atmosphere, where students actively engage in the course through online discussions and real-time interactions, showing a keen interest in the course content. Compared to traditional teaching methods, Rain Classroom has advantages in student participation and interaction. One teacher expressed, “I believe that in Rain Classroom, students can communicate with teachers and classmates anytime, anywhere through real-time interactive functions. Compared to traditional teaching methods, it provides students with greater participation space and a more convenient interactive experience, promoting the improvement of learning effectiveness.”

**Table 2:** Summary of findings and cross-case analysis of teachers’ evaluation of Rain Classroom.

Case	Findings	Similarities	Differences	Patterns/Themes
**School A**	The purpose of using the rain classroom is to improve the interaction and flexibility, the use of discussion, online quiz and resource sharing function, suggest that rain classroom can increase more interactive tools, in order to improve the students’ participation and discussion atmosphere, at the same time optimize the simulation practice experience, increase the real scene simulation, increase the voting and questionnaire tools, in order to better stimulate the participation of students. The teachers are quite satisfied with the teaching of the rain-type classroom. Students show a positive learning attitude in the course, most of them have achieved satisfactory results, rain classroom provides a more convenient and real-time interactive experience, and rain classroom has a significant advantage in promoting students’ learning participation and improving the learning effect.	Teachers all agreed that the rain classroom use effect is more satisfactory, the classroom atmosphere is better, the students’ grades and performance are better, the attitude is also very good.	Each teacher gave different suggestions, but School A suggested adding the simulation of real scenes, and adding voting and questionnaire survey tools. School B Suggestions for further improving personalized guidance and feedback functions to provide more online resources. School C It is recommended to strengthen the data analysis and evaluation function to provide a more comprehensive study data analysis report. School D It is suggested that Rain Classroom provide more practice case sharing platforms.	General Satisfaction Unique Suggestions by School *School A: Simulation of Real Scenes and Interactive Tools* *School B: Personalized Guidance and Feedback Functions* *School C: Strengthening Data Analysis and Evaluation* *School D: More Practice Case Sharing Platforms* Common Thread: Continuous Improvement and Enrichment
**School B**	Use the rain classroom in order to improve the students ‘practice and participation ability, significantly improve the students’ learning effect, the favorite function is personalized guidance, students can watch operation video in advance, field operation, said very satisfied with the use of rain classroom, but requires students to have certain self-discipline and self-study ability, suggest to further improve the personalized guidance and feedback function, to provide more online resources. The two teachers were very satisfied with the use of the rain classroom, believing that the platform has achieved positive results in improving students’ learning effect and promoting learning interaction, and that the classroom atmosphere is usually full of vitality and interaction. In the “rain classroom”, the students’ overall academic performance and performance are better.			
**School C**	Teachers often use online testing and evaluation, especially the homework release and correction function, to make students more actively engaged through real-time interaction and discussion, and the classroom atmosphere is usually very active with enthusiasm and enthusiasm. I was very satisfied with the teaching of the rain class. Teachers need to have the ability to design online courses and guide students to study independently. The teacher suggested strengthening the data analysis and evaluation function, providing a more comprehensive learning data analysis report, strengthening the mobile support, and optimizing the mobile user experience. Students’ academic performance and performance are excellent, and this novel learning environment encourages students to participate more actively in the curriculum.			
**School D**	Both teachers expressed satisfaction with the teaching satisfaction of the rain classroom. Teachers often use rain classroom for operational training, course evaluation feedback, preview effect test and real-time interactive discussion, which helps to improve students ‘practical ability. Rain classroom plays a positive role in promoting students’ interaction and improving students’ practical ability. It is suggested that Rain classroom provide more practice case sharing platforms. Students achieve encouraging results, and the classroom atmosphere is usually very active and dynamic.			
**Discussion**	While each school presents unique suggestions, a common thread emerges across all institutions—the pursuit of continuous improvement and enrichment. Whether through the simulation of real scenes, personalized guidance, enhanced data analysis, or additional practice case sharing platforms, the collective goal is to refine the Rain Classroom model to better cater to the diverse needs of students and educators. The analysis underscores the importance of adaptability and ongoing enhancement in educational technology to foster a dynamic and effective learning environment.			

### The challenges and enablers encountered

In the implementation of hybrid learning supported by Rain Classroom nursing education, the teachers faced both technical challenges and unstable internet connectivity. One teacher highlighted the challenges that students unfamiliar with online platforms may encounter. These can be dealt with by providing technical support and training, as well as providing students with detailed guidance and resources. Another teacher raised the issue of network instability that students faced due to unreliable network coverage in different geographical locations, and suggested that students find a stable network environment in which to study, but that alternative learning methods are available in case the system fails. Comprehensive coping strategies include technical support, training, detailed guidance and the provision of alternative learning methods to ensure that students can overcome technical and network barriers to reach a smooth learning environment (Table 3).

**Table 3:** Summary of findings and cross-case analysis of the Rain Classroom challenges and enablers.

Case	Findings	Similarities	Differences	Patterns/Themes
**School A**	The challenges faced in the rain classroom are the technical challenges and unstable Internet connectivity, and the coping strategies include technical support, training, and detailed guidance.	They believe that there are some challenges in the use of rain class, and teachers believe that these challenges can be met through coping strategies.	Schools that cannot face different challenges, including internal and external factors, external factors mainly include the stability of network technology, internal factors mainly include students ‘participation, teaching resources and involvement, students’ operational ability and teaching quality, etc.	Challenges Internal Factors • *Student Participation* • *Teaching Resources and Involvement* • *Students’ Operational Ability* • *Teaching Quality* • *Balancing Online and Offline Components* • *User Preferences and Device Disparities* • *Teacher Training and Adaptation* External Factors • *Network Stability* • *Technology Access and Equity* • *Data Security and Privacy* Enablers Strengthened Network Infrastructure Comprehensive Technical Support Pedagogical Training and Resources Enhanced Interactive Features Personalized Guidance and Feedback Mechanisms Expanded Online Resources Data Analysis and Reporting Enhancements Practice Case Sharing Platforms Continuous Collaboration and Feedback Loop Flexibility in Device Compatibility
**School B**	Challenges in the rain classroom are issues of course design, teaching resource preparation and student engagement, with coping strategies include advance planning, adequate preparation of teaching resources, designing interactive activities and providing timely feedback to ensure that blended learning teaching proceeds smoothly and maintains active student engagement.			
**School C**	The challenges faced in the rain classroom are the difficult problem of students’ practical operation ability, the balance challenge of personalized teaching, and the coping strategies include combining field practice and designing simulation practice activities.			
**School D**	The challenge in the rain classroom is how to maintain timely communication and feedback in the virtual environment and ensure teaching quality and effectiveness. Coping strategies include strengthening communication and feedback mechanisms, constantly optimizing teaching through evaluation and feedback, ensuring the effective implementation of mixed learning and improving the quality of teaching.			

Through these interviews with the teachers, we have identified that Rain Classroom faces certain challenges in its implementation, including both internal and external factors. Internal factors include student participation, adequacy of teaching resources, teachers’ digital literacy, teaching quality, equipment differences and training in digital technology and usage habits. External factors include network stability, equity in accessing technology and digital security.

### The model of digital teaching with Rain Classroom

**Fig 1:**
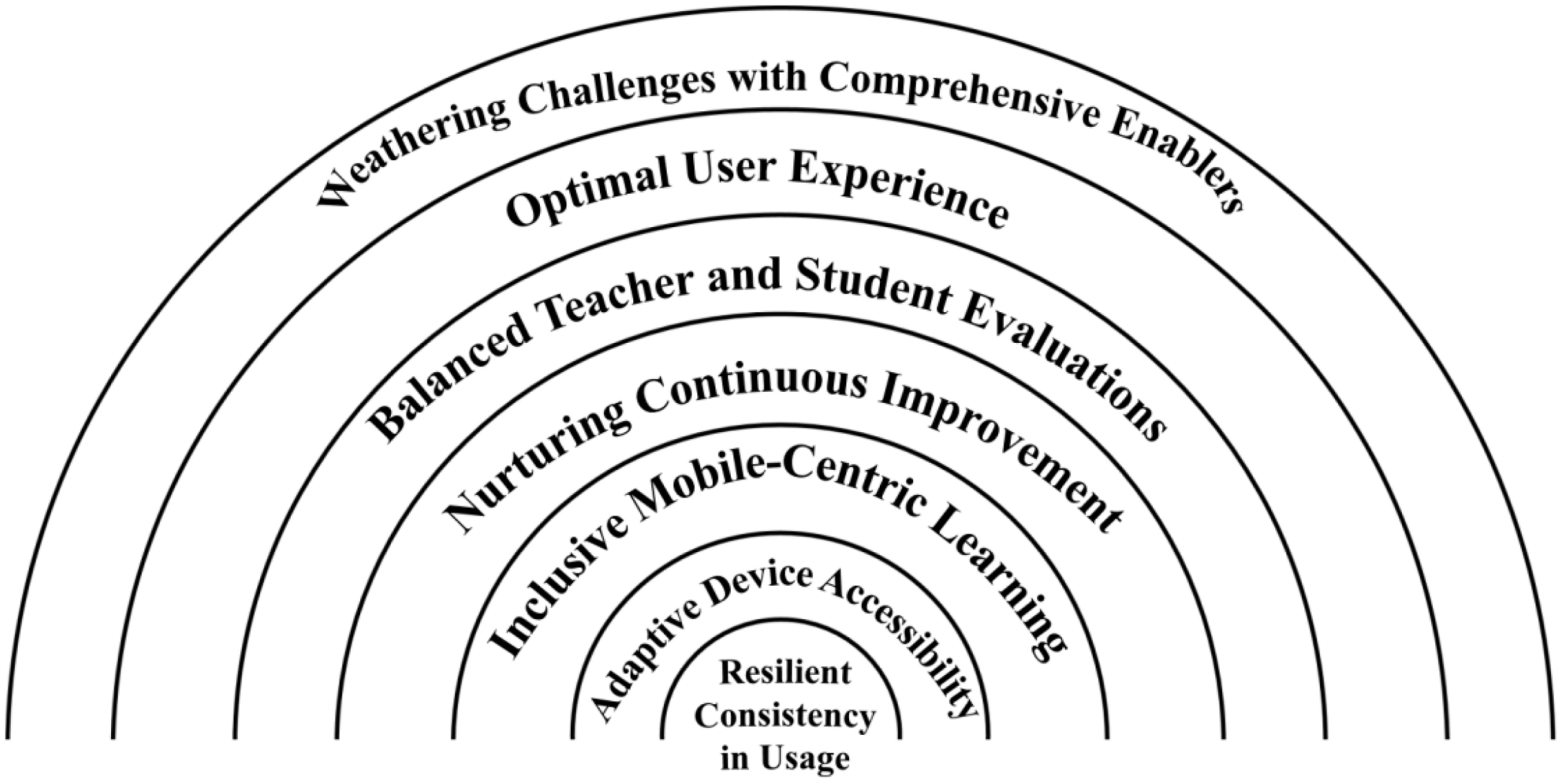
The model of sustainable implementation of Rain Classroom.

In crafting a sustainable implementation model for Rain Classroom, the Rainbow Framework (Fig 1) encapsulates key principles drawn from the study, focusing on Resilience, Adaptability, Inclusivity, Nurturing Continuous Improvement, Balanced Evaluations, Optimal User Experience and Weathering Challenges with Comprehensive Enablers.

**Resilient Consistency in Usage** (conceptual constructs). Rain Classroom endeavours to establish Resilient Consistency in Usage by cultivating a culture of regular engagement. Through targeted communication, ongoing training and recognition, the platform aims to seamlessly integrate into the daily educational routine, fostering a resilient and habitual usage pattern.

**Adaptive Device Accessibility** (conceptual constructs) Recognising the Device Preference Discrepancy, Rain Classroom commits to Adaptive Device Accessibility. The platform can be optimised for both mobile and desktop use, user-friendly interfaces can be developed, and engaging mobile-specific features can be created to cater to diverse learning preferences, thereby empowering users to adapt their usage based on individual preferences.

**Inclusive Mobile-Centric Learning** (conceptual constructs). Rain Classroom embraces mobile-centric inclusive learning, prioritizes the mobile experience, optimises mobile learning curriculum resources, enables students to learn anywhere, anytime, and promotes an inclusive learning environment.

**Nurturing Continuous Improvement** (conceptual constructs). A common thread throughout the model is Nurturing Continuous Improvement. Rain Classroom establishes a collaborative platform for schools to share best practices, unique suggestions and experiences, fostering a culture of ongoing improvement and enrichment.

**Balanced Teacher and Student Evaluations** (data-derived components). Balancing Teacher and Student Evaluations is integral. Regular surveys, feedback sessions and focus groups assess General Satisfaction, Likability of Features, and Overall Student Satisfaction. This balanced approach ensures a holistic understanding of the platform’s effectiveness and user experience.

**Optimal User Experience** (data-derived components). Focusing on Optimal User Experience, Rain Classroom will continually enhance features, such as the PPT Slide Presentation Feature. This ensures high likability and satisfaction among students, contributing to an enriching learning environment.

**Weathering Challenges with Comprehensive Enablers** (data-derived components). This is crucial for Rain Classroom’s sustainability. Strengthened Network Infrastructure, Comprehensive Technical Support, Pedagogical Training, Enhanced Interactive Features, Personalised Guidance, Expanded Online Resources, Data Analysis Enhancements, Practice Case Sharing Platforms, Continuous Collaboration and Flexibility in Device Compatibility form a robust support system to overcome challenges and facilitate sustainable implementation.

By embracing the Rainbow approach, Rain Classroom seeks to create an educational environment that is resilient, adaptive, inclusive and continuously improving, offering a diverse and enriching experience for both educators and students. This model underscores Rain Classroom’s commitment to providing an optimal and evolving platform that meets the dynamic needs of the educational community.

## Discussion

The participants in this study were teachers of all age groups that were using Rain Classroom with computers for teaching at least once a week, and had done so for at least two years. In terms of functionality, Rain Classroom covers a wide range, including online lectures, real-time interaction and discussion, simulated practice and case analysis, assignment distribution and grading, online quizzes and assessments, course evaluation and feedback, personalised guidance, operation training and pre-learning effectiveness testing. Between them, the teachers had utilised all these functions of Rain Classroom. This aligns with the current mainstream research focus in China, as the country is committed to the comprehensive popularisation and promotion of educational informatisation [18,19], reflecting its profound understanding and proactive response to the trend of educational informatisation.

Some of the teachers believed that the online discussion and interaction functions are better because they can communicate directly with students, answer questions, promote idea exchange, and make the course more interactive and engaging. This is consistent with the research by Hui Lv et al. [20], indicating that interaction is an indispensable component of information-based teaching classrooms. However, some scholars argue that interaction in information-based teaching, such as through Rain Classroom, requires active guidance and management [21], especially during online teaching rather than face-to-face instruction, to prevent the interaction content from becoming disconnected from classroom discussions.

In addition, some teachers mentioned in the interviews that the personalised guidance function was very good. Course evaluation and feedback functionality was also appreciated by teachers. They believed that through the platform’s evaluation function, students can understand their satisfaction and understanding of the course, thus adjusting teaching methods and content accordingly to improve teaching quality.

In evaluating the teaching effectiveness of Rain Classroom, all eight teachers believed that the tool had a positive impact on students’ learning outcomes and achievements, followed by a positive learning attitude. Moreover, compared to traditional teaching methods, Rain Classroom had many advantages in terms of interactivity, mainly reflected in the real-time, convenience and flexibility of interaction.

Personalised learning can formulate learning plans according to students’ learning abilities and progress, flexible learning times to accommodate the work arrangements of professional nursing students, diverse teaching resources to provide rich learning materials, practical guidance and case analysis to strengthen practical skills training, interactive learning and communities to promote communication and mutual assistance among students. Real-time feedback and evaluation help students adjust their learning strategies, update course content and ensure that students learn the latest knowledge. At the same time, the auxiliary resources and tools provided can help students better understand and master the knowledge they have learned. This is consistent with the findings of Zhang Guangbing et al. [22], indicating that personalised learning is a major advantage of Rain Classroom’s information-based teaching. It demonstrates significant benefits in enhancing students’ comprehensive abilities, effectively promoting the integration of theory and practice, and strengthening students’ professional competitiveness.

From the interviews with teachers, it can be seen that there are many factors driving the implementation of Rain Classroom. The frequency of use of Rain Classroom was unanimously recognised by the teachers at the vocational nursing schools, who believed that it improved students’ learning outcomes. In their experience, students had more positive learning attitudes, higher motivation, increased participation and greater teacher-student interaction. The teachers came to appreciate the advantages of using Rain Classroom in their teaching, including being aware of their students’ performance and attitudes during the course, and they began to use an increasing number of the tool’s many learning functions. The high satisfaction of teachers with the use of Rain Classroom will be a factor driving its implementation.

### How to address challenges

**Personalised learning:** Rain Classroom offers personalised learning capabilities, recommending relevant learning resources and content based on students’ learning abilities and needs. This caters to different students’ learning speeds and styles [23].

**Flexible learning time:** Rain Classroom provides flexible scheduling for learning, allowing vocational nursing students to choose suitable study times based on their work and life arrangements [24].

**Diverse teaching resources**: Rain Classroom typically integrates various forms of teaching resources, including text, video, audio, etc., to meet students’ needs for diverse learning resources [8].

**Practical guidance and case analysis:** Vocational nursing education emphasises the cultivation of practical skills. Rain Classroom can enhance practical skills through such methods as simulation experiments and case analysis [20].

**Online community and interactive learning:** Rain Classroom can provide a platform for communication and interaction among students. Through discussions, Q&A, etc., it facilitates mutual assistance and learning among students [24].

**Real-time feedback and assessment:** Rain Classroom offers real-time learning feedback and assessment mechanisms, helping students promptly understand their learning progress and adjust their study strategies [25].

**Updated course content**: Knowledge and techniques in vocational nursing may evolve with the times. Online education platforms may update course content promptly to ensure students learn the latest information [26].

**Supplementary Resources and Tools:** Rain Classroom can provide supplementary resources and learning tools such as course materials, teaching aids, and so on, helping students better understand and master the target knowledge [27].

With its comprehensive teaching functionalities, Rain Classroom holds potential application value across various educational contexts.

1. **Personalised student instruction**: By recording student learning data such as quiz performance and video viewing duration, Rain Classroom enables teachers to assess individual learning progress and comprehension levels. Based on this data, educators can provide tailored learning recommendations and guidance.
2. **Parent-school communication**: Rain Classroom integrates parents into class management, allowing them to stay informed about their child’s academic performance, including homework completion and exam results. This facilitates greater parental involvement in education and strengthens home-school collaboration.
3. **Online education**: Supporting live-streamed lectures, Rain Classroom allows teachers to deliver real-time explanations and presentations while enabling student interaction via bullet comments and voice connections, replicating offline classroom dynamics. Additionally, live sessions are automatically recorded for playback, letting students review content anytime to address gaps or accommodate missed sessions.
4. **Vocational training:** For corporate scenarios like new employee onboarding or skill enhancement, Rain Classroom delivers customised training courses and materials tailored to job-specific requirements and individual skill levels, improving relevance and efficacy. Its assessment features (e.g., exams, assignments) evaluate trainees’ progress, identifying knowledge or skill deficiencies to inform subsequent training adjustments.
5. **Flexible learning for professionals:** Working professionals can utilise fragmented time to access course content anytime, anywhere, completing assignments and exams to advance their knowledge and skills. The platform’s discussion forums foster learning communities where participants exchange insights, share experiences, and mutually support one another, cultivating a collaborative learning environment.

Thus, owing to its versatile functionalities, Rain Classroom demonstrates significant utility across diverse educational settings.

This section of the study aims to present tangible and actionable recommendations and suggestions which are based on the minor and major findings of the study. Based on the findings, the researcher therefore recommends the following:

1. **Recommendation to the teacher**: Make students’ aware of the importance of previewing material and cultivate their problem-solving abilities. Teachers can guide students to develop good preview habits and improve their understanding and mastery of the course content [28]. At the same time, students should also be encouraged to actively participate in discussions to improve their problem-solving skills and critical thinking [29].
2. **Recommendation to the school and teacher:** Further tap the potential of interactive functions. Schools and teachers can more flexibly use the online discussion and interactive functions of Rain Classroom in the course, to promote communication and interaction between teachers and students, thereby improving the effect of learning [30].
3. **Recommendation to the teacher:** Strengthen personalised learning and practice guidance. According to the learning needs of different students, personalised learning resources and guidance can be provided through Rain Classroom, and the function of practice guidance and case analysis can be strengthened to help students combine theoretical knowledge with practice [31].
4. **Recommendation to the school:** Continuous update and optimisation of curriculum content. Schools should regularly review and update the curriculum content provided by Rain Classroom to ensure that it is consistent with the latest teaching requirements and industry development [32].
5. **Recommendation to the school:** Improve teachers’ technical application ability. In order to make better use of the Rain Classroom teaching platform, schools should provide teachers with corresponding technical training and support to improve their ability in online teaching [33].
6. **Recommendation to the school**: Comprehensive evaluation of the learning results. Schools should evaluate the results of students’ learning in various ways, including classroom tests, homework, practical operations, etc., so as to fully understand the status of students’ learning [34]. Strengthen students’ problem-solving ability and critical thinking cultivation [35].

## Conclusion

This study focuses on the application of Rain Classroom in nursing education, and this article systematically summarises the significant advantages of its digital teaching in terms of convenience, interactivity and diversified learning functions. The Rainbow Framework for digital teaching and learning was constructed based on the Rainbow Theory Model and the core practice elements were extracted through multi-case empirical studies. The framework reveals universal principles and models for the integration of digital tools, provides key theoretical guidance and practical blueprints for the implementation of the Rain Classroom and similar tools, enhances the effectiveness of teaching and learning, and provides valuable theoretical references for the digital transformation of education.

### Limitations

However, there are some limitations of this study. First, since this study’s sample size was limited to only eight participants from four schools in China and confined to nursing vocational education, the sample scope is relatively narrow. It cannot represent the usage of Rain Classroom in other regions of China, other disciplines or different educational levels such as Master’s and Doctoral programmes. Future research should further expand the sample size, including broader or more diverse samples, to enrich the study findings.

Second, the assessment in this study was mainly based on teachers’ self-reports and did not fully incorporate students’ feedback on Rain Classroom, which affected the multidimensionality of the assessment. In the future, student perspectives, such as questionnaires, focus group interviews or data from learning analytics platforms can be incorporated to obtain direct feedback from students, enriching assessment perspectives and enabling multidimensional validation.

Third, direct observation of the actual use of Rain Classroom has not been included in this study. Future research could incorporate classroom observations (video analyses, field notes, etc.) or design observation-based action research to examine the specific processes and challenges of the platform’s use.

## Supporting information

S1 File Analysis procedure. (DOC)

S2 File Minimal data set. (DOC)
