# Supplementary material for: Emerging horizons: A Rainbow Model for the sustainable implementation of Rain Classroom in vocational nursing education
Source: PLoS One. 2025 Sep 29;20(9):e0331848. doi: 10.1371/journal.pone.0331848 (PMC12478921; doi:10.1371/journal.pone.0331848)
Supplement: S1 File — (DOC) [file pone.0331848.s001.doc]

**Table 1 ：Extent of Utilization of Rain Classroom among Teachers**

**Table 1.1 ：Case No. 1 (School A)**

| **Methods** | **Results/Findings** |
| --- | --- |
| Observation | In this interview, it can be seen that teachers of various age groups have used Rain Classroom for teaching, with usage periods of over 2 years, teaching at least once a week, and using computers for teaching. |
| Interview | Two teachers have teaching experiences of 13 and 18 years, respectively. They have been using Rain Classroom for 3 and 2 years, with a frequency of more than 1 session per week. Both teachers utilize computers for teaching, focusing on the subjects of Basic Nursing and Health Assessment. One teacher gave an online lecture through the rain classroom, and used the rain classroom platform to provide real-time or pre-recorded lectures to introduce the basic theories and practical skills of nursing science. Another person used the rain classroom for many teaching activities, including class lectures, group discussions, practical demonstrations, and homework assignments. They believe that the purpose of using rain classroom is mainly to enhance the interactivity and flexibility of teaching, while providing high-quality online nursing education resources. |

**Table 1.2 ：Case No. 2 (School B)**

| **Methods** | **Results/Findings** |
| --- | --- |
| Observation | In this interview, it can be seen that teachers of various age groups have used Rain Classroom for teaching, with usage periods of over 2 years, teaching at least once a week, and using computers for teaching，the function of the rain classroom is basically fully used. |
| Interview | Two teachers have teaching experiences of 4 and 18 years, respectively. They have been using Rain Classroom for 2 and 3 years, with a frequency of per week. Both teachers utilize computers for teaching, focusing on the subjects of Basic Nursing and primarily internal medicine nursing. The two teachers carry out a variety of teaching activities through the rain classroom, including class lectures, group discussions, practical simulation and online homework. They believe that the purpose of using rain classroom is mainly to provide students with opportunities to interact with professional educators to solve their problems and doubts in the learning process, to enhance classroom interaction and student engagement, and also to better demonstrate the actual nursing operations. |

**Table 1.3 ：Case No. 3 (School C)**

| **Methods** | **Results/Findings** |
| --- | --- |
| Observation | In this interview, it can be seen that teachers of various age groups have used Rain Classroom for teaching, with usage periods of over 2 years, teaching at least once a week, and using computers for teaching，the function of the rain classroom is basically fully used. |
| Interview | Two teachers have teaching experiences of 17 and 23 years, respectively. They have been using Rain Classroom for 2 and 3 years, with a frequency of per week. Both teachers utilize computers for teaching, focusing on the subjects of Basic Nursing and primarily internal medicine nursing. The two teachers carry out a variety of teaching activities through the rain classroom, including class lectures, group discussions, practical simulation and online homework. They believe that the purpose of using rain classroom is mainly to provide simulation practices and case studies to help students apply theoretical knowledge to practical nursing scenarios, while providing online quizzes and assessments to help students test their learning outcomes and understanding. |

**Table 1.4 ：Case No. 4 (School D)**

| **Methods** | **Results/Findings** |
| --- | --- |
| Observation | In this interview, it can be seen that teachers of various age groups have used Rain Classroom for teaching, with usage periods of 3 years, teaching at least once a week, and using computers for teaching，the function of the rain classroom is basically fully used. |
| Interview | Two teachers have teaching experiences of 19 and 28 years, respectively. They have been using Rain Classroom for 3 years, with a frequency of per week. Both teachers utilize computers for teaching, focusing on the subjects of Obstetrics and gynecology nursing and Pediatric Nursing.The two teachers carry out a variety of teaching activities through the rain classroom, including class lectures, group discussions, practical simulation and online homework. They believe that the purpose of using rain classroom is mainly to provide a flexible learning environment for students with different learning needs and rhythms to promote personalized learning experience, while providing education through online platforms, providing a way to obtain high-quality nursing education for students who are unable to participate in traditional classroom teaching. |

**Table 2：Extent of Utilization of Rain Classroom among Students**

**Table 2.1 ：Case No. 1 (School A)**

| **Methods** | **Results/Findings** |
| --- | --- |
| **Observation** | The observed trends highlight the sustained and diverse utilization of Rain Classroom among students, with a preference for mobile learning. The platform's ability to accommodate various nursing courses and the moderate study frequency further affirm its relevance and effectiveness in supporting nursing education. |
| **Questionnaire** | A total of 29 students were surveyed, all of whom have used the Rain Classroom for learning. Among them, 7 students have used it for 1 year,16 for 2 years, and 6 for 3 years, indicating widespread usage among students, with usage periods of over a year. Regarding the courses they used, they covered a wide range, including 9 students in Medical Nursing, 3 in Surgical Nursing, 6 in Pediatric Nursing, 3 in Obstetric and Gynecological Nursing, 4 in Basic Nursing, and 4 in Health Assessment. In terms of study frequency, 8 students studied less than once a week, 15 students studied 1-3 times a week, and 6 students studied more than 3 times a week, with a general concentration of 1-3 times per week, indicating moderate usage among students. It is noteworthy that most students use their mobile phones for learning, with 12 students using phones, 10 using computers, and only 7 using tablets. |

**Table 2.2：Case No. 2 (School B)**

| **Methods** | **Results/Findings** |
| --- | --- |
| **Observation** | Rain Classroom highlights a widespread and enduring usage, with students engaging for 1 to 3 years. The diverse course selection includes Medical Nursing, Surgical Nursing, Pediatric Nursing, Obstetric and Gynecological Nursing, Basic Nursing, and Health Assessment. A moderate study frequency, with 16 students studying 1-3 times a week, is observed, reflecting a consistent pattern. Notably, the majority prefer mobile phones for learning (16 students), indicating the platform's accessibility and convenience. |
| **Questionnaire** | A total of 29 students were surveyed, all of whom have used the Rain Classroom for learning. Among them, 7 students have used it for 1 year,16 for 2 years, and 6 for 3 years, indicating widespread usage among students, with usage periods of over a year. Regarding the courses they used, they covered a wide range, including 3 students in Medical Nursing, 5 in Surgical Nursing, 6 in Pediatric Nursing, 5 in Obstetric and Gynecological Nursing,2 in Basic Nursing, and 8 in Health Assessment. In terms of study frequency, 8 students studied less than once a week, 16 students studied 1-3 times a week, and 5 students studied more than 3 times a week, with a general concentration of 1-3 times per week, indicating moderate usage among students. It is noteworthy that most students use their mobile phones for learning, with 16 students using phones, 8 using computers, and only 5 using tablets. |

**Table 2.3 ：Case No. 3 (School C)**

| **Methods** | **Results/Findings** |
| --- | --- |
| **Observation** | Rain Classroom indicates widespread and consistent adoption, with varying usage durations (1 to 3 years) and diverse course selections across nursing specialties. Despite the broad coverage, the majority of students (16 out of 29) study less than once a week, suggesting a moderate usage frequency. Notably, mobile phones are the preferred device for learning, with 17 students using them, while computers and tablets have lower usage rates (9 and 3 students, respectively). Overall, the findings highlight the platform's popularity and adaptability but also suggest variations in study frequency among surveyed students. |
| **Questionnaire** | A total of 29 students were surveyed, all of whom have used the Rain Classroom for learning. Among them, 12 students have used it for 1 year,9 for 2 years, and 8 for 3 years, indicating widespread usage among students, with usage periods of over a year. Regarding the courses they used, they covered a wide range, including 6 students in Medical Nursing, 6 in Surgical Nursing, 5 in Pediatric Nursing, 5 in Obstetric and Gynecological Nursing,2 in Basic Nursing, and 4 in Health Assessment. In terms of study frequency, 16 students studied less than once a week, 5 students studied 1-3 times a week, and 8 students studied more than 3 times a week, with a general concentration of less than once a week,suggested that the use frequency is not very high. It is noteworthy that most students use their mobile phones for learning, with 17 students using phones, 9 using computers, and only 3 using tablets. |

**Table 2.4 ：Case No. 4 (School D)**

| **Methods** | **Results/Findings** |
| --- | --- |
| **Observation** | Rain Classroom indicates widespread and enduring usage, with a majority engaging for 2 years. The diverse enrollment in various nursing courses reflects the platform's versatility. Study frequency tends toward moderation, with most students studying 1-3 times per week. The notable preference for mobile phones (21 students) highlights the platform's adaptability. Overall, Rain Classroom appears to be consistently utilized and well-received among the surveyed students. |
| **Questionnaire** | A total of 29 students were surveyed, all of whom have used the Rain Classroom for learning. Among them, 5 students have used it for 1 year,22for 2 years, and 2 for 3 years, indicating widespread usage among students, with usage periods of over a year. Regarding the courses they used, they covered a wide range, including 5 students in Medical Nursing, 4 in Surgical Nursing, 8 in Pediatric Nursing, 5 in Obstetric and Gynecological Nursing,1 in Basic Nursing, and 6 in Health Assessment. In terms of study frequency, 3 students studied less than once a week, 19 students studied 1-3 times a week, and 7 students studied more than 3 times a week, with a general concentration of 1-3 times per week, indicating moderate usage among students. It is noteworthy that most students use their mobile phones for learning, with 21 students using phones, 6 using computers, and only 2 using tablets. |

***Table 3：****Summary of Findings and Cross-case Analysis of the Rain Classroom Utilization*

| **Case** | | **Findings** | **Similarities** | | **Differences** | | **Patterns/ Themes** |
| --- | --- | --- | --- | --- | --- | --- | --- |
| **Case A** | Teacher | Use Rain Classroom for 3 and 2 years, with a frequency of more than 1 session per week. Both teachers utilize computers for teaching, focusing on the subjects of Basic Nursing and Health Assessment. | Both students and teachers used the rain classroom for more than a year, and they study at least once a week | Both students and teachers used the rain classroom for more than a year, and they study at least once a week | Students used more mobile phones to study, and teachers are more inclined to choose computers for rain classroom | Students used more mobile phones to study, and teachers are more inclined to choose computers for rain classroom | Consistent usage patterns  Device Preference Discrepancy  Mobile-Centric Student Learning  Teacher Emphasis on Desktop Efficiency |
|  | Student | 7 students have used it for 1 year,16 for 2 years, and 6 for 3 years |  |  |  |  |  |
| **Case B** | Teacher | Use Rain Classroom for 2 and 3 years, with a frequency of per week. Both teachers utilize computers for teaching. | Both students and teachers use the rain class for more than a year, and they study at least once a week |  | Students use more mobile phones to study, and teachers are more inclined to choose computers for rain classes |  |  |
|  | Student | 7 students have used it for 1 year,16 for 2 years, and 6 for 3 years, |  |  | Students use more mobile phones to study, and teachers are more inclined to choose computers for rain classes |  |  |
| **Case C** | Teacher | Use Rain Classroom for 2 and 3 years, with a frequency of per week. Both teachers utilize computers for teaching. | Both students and teachers use the rain class for more than a year, and they study at least once a week |  | Students use more mobile phones to study, and teachers are more inclined to choose computers for rain classes |  |  |
|  | Student | 12 students have used it for 1 year,9 for 2 years, and 8 for 3 years. |  |  | Students use more mobile phones to study, and teachers are more inclined to choose computers for rain classes |  |  |
| **Case D** | Teacher | Use Rain Classroom for 3 years, with a frequency of per week. Both teachers utilize computers for teaching,. | Both students and teachers use the rain class for more than a year, and they study at least once a week |  | Students use more mobile phones to study, and teachers are more inclined to choose computers for rain classes |  |  |
|  | Student | 5 students have used it for 1 year,22for 2 years, and 2 for 3 years, |  |  |  |  |  |

**Table 4：Evaluation of Teachers in Rain Classroom according to Functionalities**

**Table 4.1: Case No. 1 (School A)**

| **Methods** | **Results/Findings** |
| --- | --- |
| **Observation** | The Rain classroom creates a positive and interactive classroom atmosphere. Students are actively engaged in the course in this virtual environment through online discussion and real-time interaction. They show a good learning state and show a strong interest in the course content. In the teaching environment of the rain classroom, the classroom atmosphere is very active and warm. Students fully express their views and thoughts through online discussion and real-time interaction. They maintain a good learning state and show a positive learning attitude. |
| **Interview** | Overall, both teachers emphasized the purpose of enhancing interactivity and flexibility when using the rain classroom for nursing education. In terms of teaching activities, they introduced the theoretical and practical skills of nursing through online lectures, real-time interaction and simulated practice. When implementing mixed learning, both teachers faced technical problems, but solved the problem of blocked learning for students by providing technical support and training.  In terms of function use, both teachers focused on the discussion section, online quizzes and resource sharing functions to facilitate student interaction, assess learning progress and facilitate the sharing of teaching materials. They all particularly like online discussion and interactive features, which they help to communicate directly with students and promote the exchange of ideas.  As for the improvement suggestions, they all hope that Rain Classroom can further enhance the interactive function, such as adding more interactive tools to enhance students' participation and discussion atmosphere. One of the teachers also mentioned optimizing the simulation practice experience, increasing the simulation of real scenes, and providing more practical opportunities to better help students cope with the challenges in practical nursing work. |

**Table 4.2: Case No. 2 (School B)**

| **Methods** | **Results/Findings** |
| --- | --- |
| **Observation** | The teaching environment of the rain classroom usually creates a positive and interactive atmosphere. Students actively participate in the course through online discussion and real-time interaction. They maintain a good learning status and show a high degree of concentration and enthusiasm for learning. In the teaching environment of the rain classroom, the classroom atmosphere is very active and warm. Students fully express their views and thoughts through online discussion and real-time interaction. They maintain a good learning state and show a positive learning attitude. |
| **Interview** | Overall, the two teachers jointly emphasized the importance of simulation practice and personalized guidance in the rain classroom instruction. They let students practice nursing skills in a virtual environment through a video or interactive simulation of simulated practices. My favorite function is personalized guidance, in which students can watch the operation videos in advance, conduct on-site operation, and get real-time comments and guidance.  In implementing mixed learning, teachers believe that the advantage of rain classroom is to improve students 'practical ability and participation, but also pointed out the challenges that may involve students' self-discipline and technical training. Suggestions for improvement include providing more online resources and expanding the teaching resource base to help students have a more comprehensive understanding of nursing knowledge.  In general, both teachers expressed high satisfaction with the use of rain classroom, believing that it has played a positive role in nursing education and improved students' learning effect and practical operation ability. As for the improvement suggestions, teachers propose to further improve the personalized guidance and feedback functions to support students' learning needs more targeted. In general, teachers hold a positive evaluation of the rain classroom, believing that it plays a role in promoting students' learning in nursing education. |

**Table 4.3: Case No. 3 (School C)**

| **Methods** | **Results/Findings** |
| --- | --- |
| **Observation** | In the rain classroom, the classroom atmosphere is usually very active. Students are actively involved in the course through real-time interaction and discussion. They maintain a good learning state and show a positive attitude towards learning. Rain classroom atmosphere in the classroom teaching environment is often very warm and positive. Students actively participate in the course through real-time interaction and discussion. They maintain a good learning state and show their thirst for and love for knowledge. |
| **Interview** | Overall, the two teachers in the rain classroom teaching make full use of the platform to provide a number of functions, one of which mainly focus on online test and evaluation, especially like homework release and correcting function, emphasize these tools help to timely evaluate students' learning outcomes, provide specific advice and feedback, enhance the teaching flexibility and interactivity.  Another teacher mainly uses the functions of course evaluation and feedback, personalized guidance, online testing and resource sharing. She especially likes the function of resource sharing. By sharing rich teaching resources, such as course syllabus, reference materials, video links, etc. In terms of the improvement of students' learning effect, the teacher believed that Rain Classroom made students more actively involved through real-time interaction and discussion, mentioning a specific case discussion that demonstrated that students learned practical nursing skills during the interaction. In the analysis of the advantages and disadvantages of mixed learning, the advantages include improving learning flexibility and autonomy, and the disadvantages involve device dependence. Suggestions for improvement involve enhanced mobile support to improve students' user experience on a phone or tablet. In general, the teacher was very satisfied with the use of the rain classroom, and believed that it had played a positive role in improving the teaching effect. |

**Table 4.4: Case No. 4 (School D)**

| **Methods** | **Results/Findings** |
| --- | --- |
| **Observation** | It was observed that rain classroom had a positive impact on the learning effect of students through real-time interaction and discussion, making students more actively involved, asking valuable questions, and further understanding and application of professional knowledge in case analysis. |
| **Interview** | In the rain classroom teaching, both teachers make full use of the functions of the platform, mainly focusing on the operation training, course evaluation feedback, preview effect test and real-time interactive discussion. These functions help to arrange learning in advance, evaluate students' level, realize interactive communication, and enhance the flexibility and interactivity of teaching.  When using the rain classroom for mixed learning, teachers agreed that this teaching model helped to improve students' practical abilities. However, it was also mentioned that some technical training might be needed to ensure that all students could use the platform smoothly. Suggestions for improvement include providing more practice case-sharing platforms, promoting students and teachers to share successful experiences and lessons, while strengthening teacher training and support, and providing teachers with more training resources on online teaching and instructional design to help them better use the rain classroom for nursing education. |

**Table 5:Evaluation of Teachers on Rain Classroom according to Teaching Satisfaction**

**Table 5.1: Case No. 1 (School A)**

| **Methods** | **Results/Findings** |
| --- | --- |
| **Observation** | Through real-time interaction in teaching, students can participate more actively, solve doubts, share experience, and have a more comprehensive understanding of professional knowledge。 |
| **Interview** | The two teachers were generally more satisfied with the teaching of the rain classroom. They agreed that the rain classroom provided rich features that meet teaching needs, especially in promoting real-time interaction and discussion. In terms of students' learning effect, the simulation practice and case analysis of rain classroom are recognized, which helps students to conduct practical operation and skill training in the virtual environment.  In mixed learning, the two teachers agreed that rain class supported personalized learning paths and feedback, but also noted that there may be technical requirements for some students. It is suggested to enhance the interactive function of rain classroom and improve the real-time interaction and discussion function, such as adding voting and questionnaire survey tools to better stimulate students' participation.  In addition, the two teachers put forward suggestions for the improvement of the simulated practice experience of the rain class, and suggested adding more real scenarios and providing more practical operation opportunities, so as to help students better cope with the challenges in the actual nursing work.  In general, the two teachers are satisfied with the use of the rain classroom, believing that the platform has played a positive role in improving teaching results, promoting students' participation and cultivating practical ability. Suggestions for improvement mainly focus on enhancing the interactive functions and optimizing the simulation practice experience.。 |

**Table 5.2: Case No. 2 (School B)**

| **Methods** | **Results/Findings** |
| --- | --- |
| **Observation** | Through online discussion and interaction, students are more actively involved in the course, which promotes knowledge sharing and deepening, and increases the interest of learning. |
| **Interview** | The two teachers were generally more satisfied with the teaching of the rain classroom. They believe that the rain classroom provides flexibility and allows students to choose to participate in courses based on their own schedule, making learning more autonomous and convenient. In terms of learning effect, the two teachers agreed that the use of rain classroom significantly improved the learning effect of students. In terms of mixed learning, the two teachers agreed that rain classroom supports simulation practice, effectively improving students' practical ability and preparing for future field practice. However, it is also pointed out that the mixed learning mode requires students to have certain self-discipline and self-learning ability, which may have higher requirements for students. Suggestions for improvement mainly focus on providing more online resources, enriching students 'learning resources, and further improving personalized guidance and feedback functions, and providing more targeted suggestions and guidance according to students' learning performance and needs.  In general, the two teachers are very satisfied with the use of the rain classroom, and believe that the platform has achieved positive results in improving students' learning effect and promoting learning interaction. The suggestions focus on enriching learning resources and further improving personalized guidance. |

**Table 5.3: Case No. 3 (School C)**

| **Methods** | **Results/Findings** |
| --- | --- |
| **Observation** | The students were all very active. |
| **Interview** | Both teachers expressed a high degree of satisfaction with the teaching of the rain class. They believe that the rich functions and resources of the rain classroom make the teaching more vivid and interesting. Through online testing and evaluation, students can understand the learning situation in time, and provide targeted feedback and guidance, which effectively promotes the learning effect of students.  In terms of mixed learning, rain classroom provides the function of simulation practice, which effectively improves the students' practical ability, which has been unanimously recognized by the two teachers. However, it is also pointed out that teachers need to have the ability to design online courses and guide students to independent learning, which puts forward some requirements for improving the quality of teaching.  Improvement suggestions mainly focus on strengthening the data analysis and evaluation functions, and providing a more comprehensive study data analysis report, so that teachers can better understand the students' learning situation and further adjust their teaching strategies. Another teacher suggested enhancing mobile support and optimizing the mobile user experience, to ensure that students can easily learn and interact on mobile phones or tablets.  In general, the two teachers highly recognized the positive role of rain classroom in improving students' learning effect and enhancing practical ability, and also put forward some beneficial suggestions for improvement, which provided strong feedback for the further development of rain classroom. |

**Table 5.4: Case No. 4 (School D)**

| **Methods** | **Results/Findings** |
| --- | --- |
| **Observation** | It was observed that the Rain Classroom had a positive impact on student learning outcomes, and through real-time interaction and discussion, students were more actively involved and asked valuable questions. For example, in the discussion of pain assessment and management, the students gained insight into the principles and methods of pain management to help with future practices. |
| **Interview** | Both teachers expressed satisfaction with the teaching satisfaction of the Rain Classroom, believing that the rain class is very convenient to use in the case of no face-to-face teaching.  The advantages of using the rain classroom as a teaching aid in nursing education to implement mixed learning were highlighted as being able to enhance practical competence, but also mentioned that some technical training may be needed to ensure smooth use by all students.  Improvement suggestions mainly include providing more practice case sharing platforms, so that students and teachers can share successful experiences and lessons, and promoting the combination of practice and theory. On the other hand, it is suggested to strengthen teacher training and support and provide teachers with more training resources on online teaching and teaching design to help them make better use of the rain classroom for nursing education.  In general, the two teachers 'views emphasize the positive role of the rain classroom in promoting students' interaction and improving their practical ability, while also focusing on the possible technical training needs and the desire to improve teacher support. |

**Table 6：Evaluation of Teachers on Rain Classroom according to Assessment of Teaching Effectiveness**

**Table 6.1：Case No. 1 (School A)**

| **Methods** | **Results/Findings** |
| --- | --- |
| **Observation** | The Rain classroom creates a positive and interactive classroom atmosphere. Students are actively engaged in the course in this virtual environment through online discussion and real-time interaction. They show a good learning state and show a strong interest in the course content. In the teaching environment of the rain classroom, the classroom atmosphere is usually full of energy and interaction. Students actively participate in the course through real-time interaction and discussion in the virtual space. They show a good learning state, showing a high degree of concentration and desire for knowledge. |
| **Interview** | The two teachers made positive comments on the academic performance and performance of the students in the Rain Classroom. They observed that students showed a positive learning attitude in the course, and through real-time interaction and discussion, students were more actively involved in the course. In terms of grades, most of the students have achieved satisfactory results, reflecting their efforts and learning results. Compared with traditional teaching methods, rain classroom provides a more convenient and real-time interactive experience, and promotes a more flexible and positive interaction between students, teachers and students. In the teaching environment of the rain classroom, the classroom atmosphere is full of vitality and interaction, and the students show a high degree of concentration and desire for knowledge, and show a strong interest in the course content. In general, the rain classroom shows significant advantages in promoting students' learning participation and improving their learning effect. |

**Table 6.2：Case No. 2 (School B)**

| **Methods** | **Results/Findings** |
| --- | --- |
| **Observation** | The classroom atmosphere is very active and warm. Students fully express their views and thoughts through online discussion and real-time interaction. They maintain a good learning state and show a positive learning attitude. The teaching environment of the rain classroom usually creates a positive and interactive atmosphere. Students actively participate in the course through online discussion and real-time interaction. They maintain a good learning status and show a high degree of concentration and enthusiasm for learning. |
| **Interview** | In the "Rain classroom", the students' overall learning performance and performance are good. Many students showed a positive learning attitude in the course, participating in discussions and completing assignments. In terms of grades, most of the students have achieved satisfactory results, reflecting their hard work and learning results. Compared with traditional teaching methods, rain classroom has significant differences in students' participation and interaction. Through the real-time interaction function, students can ask questions and participate in discussions at any time during the course, and maintain close contact with teachers and classmates. This interactivity greatly promotes the students' participation and learning effect. In the teaching environment of the rain classroom, the classroom atmosphere is usually full of energy and interaction. Students actively participate in the course through real-time interaction and discussion in the virtual space. They show a good learning state, showing a high degree of concentration and desire for knowledge. In general, rain classroom provides students with high-quality learning experience, promotes students' active participation and the improvement of learning effect. |

**Table 6.3：Case No. 3 (School C)**

| **Methods** | **Results/Findings** |
| --- | --- |
| **Observation** | In Rain classroom, the atmosphere is usually very active. Students actively participate in the course through real-time interaction and discussion. They maintain a good state of learning and show a positive attitude towards learning. The classroom atmosphere in the rain classroom teaching environment is often very warm and positive. Students actively participate in the course through real-time interaction and discussion. They maintain a good state of study, showing a thirst for knowledge and love. In rain classes, the atmosphere is usually very active. Students actively participate in the course through real-time interaction and discussion. They maintain a good state of learning and show a positive attitude towards learning. The classroom atmosphere in the rain classroom teaching environment is often very warm and positive. Students actively participate in the course through real-time interaction and discussion. They maintain a good state of study, showing a thirst for knowledge and love. |
| **Interview** | In the "Rain classroom", the students' learning performance and performance are excellent. The two teachers jointly pointed out that the students showed a positive attitude towards learning in the course, and showed a strong motivation for learning through real-time interaction, participating in discussions and completing assignments. Many students achieved satisfactory and even encouraging results in quizzes and assignments, reflecting their serious study and effort in the curriculum.  Compared with traditional teaching methods, rain classroom is significantly different in terms of students' participation and interaction. The real-time interaction enables students to ask questions more freely, participate in discussions, and interact with teachers and classmates more closely and efficiently. This novel learning environment prompted students to participate more actively in the curriculum.  In the teaching environment of the rain classroom, the classroom atmosphere is usually very active, full of enthusiasm and enthusiasm. Through real-time interaction and discussion, the students actively participated in the course, maintained a good learning state, and showed their desire for and love for academic knowledge. In general, the rain classroom provides a good learning platform for students and stimulates students' interest and motivation in learning. |

**Table 6.4：Case No. 4 (School D)**

| **Methods** | **Results/Findings** |
| --- | --- |
| **Observation** | The classroom atmosphere is very warm and energetic, they maintain a good learning state, showing a high commitment to learning. |
| **Interview** | In the "Rain classroom", students' academic performance and performance have been positively evaluated. Both teachers agreed that students performed well in the course, showing strong motivation through active participation, real-time interaction and homework completion. In grades, the students achieved encouraging results reflecting their serious effort and effort in the course.  Compared with traditional teaching methods, rain classroom brings significant differences in students' participation and interaction. The real-time interaction function provides a greater space for students to participate, allowing them to communicate with teachers and classmates anytime and anywhere. This convenient interactive experience promotes the effect of learning and enables students to participate in class discussions more flexibly.  In the teaching environment of the rain classroom, the classroom atmosphere is usually very active and dynamic. Through real-time interaction and discussion, the students actively participate in the course, maintain a good learning state, and show a strong thirst for knowledge. In general, Rain classroom provides a positive learning platform for students and stimulates their academic interest and learning motivation. |
