# Supplementary material for: Emerging horizons: A Rainbow Model for the sustainable implementation of Rain Classroom in vocational nursing education
Source: PLoS One. 2025 Sep 29;20(9):e0331848. doi: 10.1371/journal.pone.0331848 (PMC12478921; doi:10.1371/journal.pone.0331848)
Supplement: S2 File — (DOC) [file pone.0331848.s002.doc]

***Part Ⅲ: Teacher Interview***

Dear Teacher,

Thank you very much for taking the time to participate in this interview. In order to gain a deeper understanding of the application of blended learning using Rain Classroom in vocational nursing education, this study has designed the following questions. Your answers will greatly assist this research. Thank you for your cooperation.

*Part 1: Personal Information*

a. Name:

b. Age:

c. Education level:

d. Educational background:

e. Duration of using Rain Classroom:

*Part 2: Usage of Rain Classroom*

a. What are your purposes and motivations for using Rain Classroom in vocational nursing education?

b. What teaching activities do you conduct using Rain Classroom in vocational nursing education? (e.g., classroom lectures, group discussions, assignment assignments, etc.)

c. In your opinion, what are the advantages and conveniences of using Rain Classroom for teaching in vocational nursing education?

d. What challenges have you encountered in implementing blended learning supported by Rain Classroom vocational nursing education? From your perspective, how can these challenges be addressed? What teaching methods do you usually employ in your actual teaching?

*Part 3: Functionalities*

a. Which functionalities have you used in Rain Classroom teaching?

b. How have these functionalities assisted your teaching?

c. What is your favorite Rain Classroom functionality, and why?

*Part4: Teaching Satisfaction*

a. How satisfied are you with Rain Classroom in vocational nursing education? Please explain your reasons.

b. How do you think Rain Classroom has affected student engagement and interactivity in the teaching process?

c. Do you believe that Rain Classroom has improved students' learning outcomes? Please provide examples.

d. How do you think Rain Classroom has influenced students' learning motivation and interest?

e. What are the advantages and disadvantages of implementing blended learning using Rain Classroom as a teaching aid in vocational nursing education?

f. Do you have any suggestions for improving Rain Classroom?

*Part 5: Assessment of Teaching Effectiveness*

a. How do you assess students' learning performance and grades in Rain Classroom?

b. Do you think students' engagement and interactivity are higher in Rain Classroom compared to traditional teaching methods? Why?

c. What are the classroom atmosphere and students' learning status like in a Rain Classroom teaching setting? Have there been any changes in the class over a semester?

*Part 6: Other Opinions and Suggestions*

a. Do you have any additional comments or insights regarding your experience and perception of using Rain Classroom in vocational nursing education?

b. Do you think the teaching model using Rain Classroom is applicable to all subjects and courses?

c. What suggestions do you have for vocational nursing education based on Rain Classroom? Would you be willing to continue using the blended learning model supported by Rain Classroom in the next semester?

***Part Ⅳ Classroom Teaching Evaluation Questionnaire***

Dear students,

This is a questionnaire regarding the blended learning model supported by Rain Classroom in nursing education. After a period of learning, we believe that you have gained some insights into this learning model. This questionnaire will help teachers improve their teaching and enhance the effectiveness of blended learning. Please read it carefully and select your answers based on your personal experiences. Your feedback is highly important to me. This survey will be conducted anonymously, and the provided information will be kept strictly confidential. Thank you very much for your participation and completion!

| dimensionality | problem | conform | common | inconformity |
| --- | --- | --- | --- | --- |
| Degree of Likability for Rain Classroom Features | Likability of PPT slide presentation feature |  |  |  |
|  | Likability of pre-class attendance feature |  |  |  |
|  | Likability of pre-class quiz feature |  |  |  |
|  | Appreciation of the "Confusion" and "Favorites" feature |  |  |  |
|  | Likability of reviewing classroom content |  |  |  |
|  | Likability of post-class learning test for review and reinforcement |  |  |  |
| Acceptance and Satisfaction of Hybrid Learning Model Supported by Rain Classroom | I like using this hybrid learning model in the classroom |  |  |  |
|  | It allows me to familiarize myself with class examples in advance, and I look forward to classroom teaching |  |  |  |
|  | It makes me feel that the learning difficulty of the course has decreased and it has increased my interest in learning |  |  |  |
|  | I am willing for the teacher to continue using the student-centered hybrid teaching model in the classroom |  |  |  |
|  | I like the feature of Rain Classroom platform that provides a summary of post-class learning data reports |  |  |  |
| Attitude towards Learning and Study Habits | I feel more proactive in actively listening to the class and responding to the teacher's questions. |  |  |  |
|  | I actively engage in independent learning of the resources provided by the teacher before class, developing a habit of proactive previewing. |  |  |  |
|  | During class, I actively engage in discussions and provide constructive feedback on others' contributions and achievements. |  |  |  |
|  | I am willing to proactively ask the teacher questions, leading to increased participation in classroom activities. |  |  |  |
|  | After acquiring knowledge, I feel a sense of personal achievement and become more motivated to complete learning tasks. |  |  |  |
| Learning Achievements and Learning Effectiveness | In the process of blended learning, I actively participate in discussions and collaborative group work, which improves my focus and concentration. |  |  |  |
|  | By reviewing the materials multiple times, I have become proficient in using software tools, enhancing my problem-solving and critical thinking abilities. |  |  |  |
|  | I actively engage in self-assessment and peer evaluation, learning from others and incorporating feedback from teachers and classmates, which broadens my perspectives. |  |  |  |
|  | I am able to complete individual and group project cases. |  |  |  |
|  | The classroom environment is lively, and my social communication and teamwork skills have improved. |  |  |  |

***Part V Reliability and Validity***

**QUESTIONNAIRE ESTABLISHING THE CONTENT VALIDITY OF THE INSTRUMENT**

Name:________________________________Position/Designation:___________________

Directions: Please read all the directions and items in the research instrument and rate it as a whole using the scale below. Kindly put a ( ) mark on the space which corresponds to your evaluation opposite each indicator to determine the validity.

Weight Level

3 Highly Valid (HV). No flaws observed; nothing more is needed to make it

better

2 Valid (V). Some flaws are observed; the overall usefulness of the items is

slightly diminished.

1 Not Valid (NV). Major Revision of the item is needed to make it useful.

| **Indicators** | **HV**  **3** | **V**  **2** | **NV**  **1** |
| --- | --- | --- | --- |
| 1. The directions stated in all subsections of  the instrument are clear. | √ |  |  |
| 2. Each of the items is clearly stated. | √ |  |  |
| 3. Each of the items is readable. | √ |  |  |
| 4. Enough space is provided to avoid crowding among items. | √ |  |  |
| 5. The instrument is comprehensive (it covers areas that are important in the study). | √ |  |  |
| 6. Each of the items is focused on one particular thought or idea. | √ |  |  |
| 7. The items are objective (the responses to be elicited are neither biased nor reactive). | √ |  |  |
| 8. The items are systematically arranged according to desirable sequence. | √ |  |  |
| 9. The items do not overlap with one another. | √ |  |  |
| 10. The items are formulated in accordance to the explicit/implicit objectives. | √ |  |  |

***Part Ⅵ Pre-Interview transcripts***

We are conducting a case study on blended learning in rain classrooms and their satisfaction with the sustainability model, aiming to understand students' attitudes and opinions about blended learning. We cordially invite you to participate in our study and thank you very much for taking part in this interview within your busy schedule. In order to gain a deeper understanding of the use of blended learning in nursing education in the rain classroom, the following questions have been designed for this study:Your answers will greatly assist this study. We will make sure that the content of your conversation is confidential and your personal information will not be disclosed to anyone. Do you agree to this?

I'm today's interview investigator and I've contacted you before, so let's get started.

Interviewer : Hello, thank you for participating in our study. First, please tell me your age.

Teacher interviewer (teacher): I am 42 years old.

INTERVIEW: What is your education?

Teacher: I have a master's degree in nursing.

INTERVIEW: What is your current job title?

Teacher: I am a senior lecturer.

INTERVIEW: How many years have you been an educator in the nursing field?

Teacher: I have been a nursing educator for over ten years.

INTERVIEW: The courses you teach are?

Faculty : I am responsible for teaching basic nursing theory courses and clinical practice.

INTERVIEW: So how has your experience been in using Rain Classroom? What is the frequency and length of use?

TEACHER: I started using Rain Classroom about three years ago and would use it in my classes about four to five hours a week.

INTERVIEW: Understand, next we will talk about your purpose for using the Rain Classroom in nursing education. What do you see as its main purpose?

Teacher: I think Rain Classroom can provide an online learning platform for students to access course materials and participate in discussions anytime, anywhere.

INTERVIEW: Thank you very much for your answer. Let's move on to what specific teaching activities have you done in nursing education using Rain Classroom?

Teacher: I mainly use Rain Classroom in my courses for online group discussions, posting assignments, and also uploading some supplemental materials for students' reference.

INTERVIEW: This sounds very useful. What do you see as the advantages of using Rain Classroom in nursing education?

Teacher: I think it promotes student interaction and engagement, as well as facilitates communication between teachers and students. In addition, it provides a convenient way to share teaching resources.

INTERVIEW: So what are some of the challenges you have encountered in implementing blended learning supported by Rain classroom Nursing Education? How did you address these challenges?

Teacher: One challenge is making sure that all students have smooth access to the online platform, which sometimes involves technical issues. I will conduct technology training well in advance of the course and provide technical support during the semester.

INTERVIEW: Thank you so much for sharing your experience. Next we'll talk about the specific features you've used to teach in your rain classroom. What features do you use? How do they help your teaching?

Teacher: I often use the discussion feature of Rain classroom to organize student participation in course discussions, as well as the homework feature to assign tasks and collect student work.

INTERVIEW: That sounds very useful. So, what's your favorite Rain Classroom feature? Why?

Teacher: My favorite feature is the discussion board because it allows students to think and interact outside of the course and promotes their learning.

INTERVIEW: Thank you very much for your detailed answer. Next we will talk about your satisfaction with Rain Classroom instruction. Are you satisfied with your Rain Classroom instruction? Why?

Teacher: I am more satisfied with the teaching of Rain Classroom. It facilitates my teaching a lot and makes the interaction between students and teachers smoother.

INTERVIEW: Do you think Rain Classroom has improved student learning? Please provide examples.

Teacher: Yes, I think Rain Classroom can enhance student learning. Through online discussions and assignments, students are better able to understand the course content in depth and are able to apply what they have learned in practice.

INTERVIEW: What are the advantages and disadvantages of using the rain classroom as a teaching aid to implement blended learning in nursing education?

Teachers: the advantages are that it promotes student engagement and interaction, as well as providing an easy way for teachers to manage and give feedback. Disadvantages may be that some students may not be familiar with online learning and will need some time to get used to it.

INTERVIEW: Thank you for sharing your thoughts. Next, let's talk about suggestions for improving Rain Classroom. Do you have any suggestions?

Teacher: I hope that Rain Classroom can provide more multimedia resources and more flexible course management functions, which can better meet the needs of teachers and students.

Interview: Thank you very much for your suggestion. Next, we will evaluate the performance and achievement of the students in the Rain Room. How do you evaluate the performance and achievement of the students in the Rain Room?

Teachers: Overall, the students' participation in the Rain Classroom has been very positive.

There was a marked improvement in engagement and performance. The quality of their work has also improved.

INTERVIEW: How do you think Rain Classroom compares to traditional teaching methods in terms of student engagement and interaction?

Teacher: Compared to traditional teaching methods, Rain Classroom promotes more interaction and discussion among students. Students can freely exchange opinions and ideas online.

INTERVIEW: What is the classroom climate and student learning in a rain classroom teaching environment?

Teacher: The atmosphere in the classroom was more active and congenial, and students seemed to be more engaged and more willing to ask questions and share their opinions.

INTERVIEW: Thank you very much for your detailed answers. The last two questions, do you think the Rain Classroom model of teaching is applicable to all subjects and programs?

Teacher: I think the Rain Classroom teaching model can be applied to most subjects and courses, especially those that require interaction and discussion.

INTERVIEW: Based on the Rain Classroom, what would you recommend for nursing education?

Teacher: I suggest that more emphasis be placed on practice and case studies in teaching so that students can apply their theoretical knowledge to practical nursing.

INTERVIEW: Thank you very much for your valuable comments, your participation is very important for our research. Thank you again for your time and sharing.

***Part Ⅶ Teacher Interview transcripts***

**Interview 1**

Part I: Personal Information

a. Age: :36 b. Education: Postgraduate c. Title: Associate Professor d: Years of experience: 13

e. What you teach (to say one nursing course): Basic Nursing

f. Duration and frequency of use of RainClass: I have been using RainClass for over two years and usually spend about five hours a week teaching on the platform.

Part II: Usage of Rain Classroom

a. What is the purpose of your use of the rain classroom in nursing education?

I use Rain Classroom mainly to enhance the interactivity and flexibility of teaching. It provides a convenient online learning environment for students and me.

b. What teaching and learning activities have been conducted using the rain classroom in nursing education?

I conducted online lectures in Rain Classroom, which provides real-time or prerecorded lectures on basic nursing theories, practical skills, etc. through the Rain Classroom platform.

c. What do you see as the advantages of using the rainy day classroom for teaching in nursing education?

Teaching activities such as simulation practice and case study can allow students to practice various nursing skills in a virtual environment and be fully prepared for actual clinical practice.

d. What challenges have you encountered in implementing blended learning supported by rainy day classroom nursing education? How were these challenges addressed?

The challenge I have encountered when implementing blended learning is that technological issues cause students to be hindered in their learning. Some students may not be familiar or comfortable using online educational platforms and this may hinder their learning.

To address these issues, technical support and training are provided to provide students with guidance and resources for using the Rain classroom platform to ensure that they are able to successfully engage in learning.

Part III: Functions

a. What features do you use in your rain classroom instruction? How do these features help your teaching?

I used the discussion boards, online quizzes, and resource sharing features. The discussion boards facilitated interaction among students, while the online quizzes helped me assess students' progress. The resource sharing feature facilitated the sharing and discussion of instructional materials.

b. What is your favorite feature of the Rain Classroom? Why?

My favorite feature is the online discussion and interaction. This feature allows me to communicate directly with students, answer questions, facilitate the exchange of ideas, and make the course more interactive and engaging.

Part IV: Satisfaction with Teaching

a. Are you satisfied with your teaching in the rain classroom? Why?

I am very pleased with the Rain Classroom teaching platform, which offers a wealth of features to meet my teaching needs.

b. Do you think the rain classroom has improved student learning? Please provide examples.

Yes, I think Rain Classroom has played an active role in enhancing student learning. Through real-time interactions and discussions, students are more actively engaged in the program, addressing concerns and sharing experiences. For example, we had in-depth discussions on specific cases, and students gained a more comprehensive understanding of the practice, which may not be easy to achieve in traditional teaching.

c. What are the advantages and disadvantages of using the rain classroom as a teaching aid to implement blended learning in nursing education?

RainClass supports personalized learning paths and feedback, and can provide customized teaching content and guidance based on students' learning progress and needs, promoting students' personalized growth. However, for some students, certain technical skills and equipment support may be required to use RainLearning successfully, requiring certain technical conditions.

d. Do you have any suggestions for improvements to the Rain Room?

I would like to see Rain Classroom enhanced with interactive features. To further enhance the real-time interactive and discussion features, consider adding more interactive tools such as polls and questionnaires to stimulate student participation and discussion.

Part V: Evaluation of Teaching Effectiveness

a. How would you assess the performance and achievement of the students in the Rain Room?

We noted overall good student performance and achievement in the Rain Room. Many students demonstrated a positive attitude to learning in the program, participating in discussions and completing assignments in a timely manner. In terms of grades, most students achieved satisfactory grades, reflecting their efforts and learning outcomes.

b. How do you think the rain classroom compares to traditional teaching methods in terms of student engagement and interaction?

Compared to traditional teaching methods, Rain Classroom has a significant difference in terms of student participation and interaction. With the real-time interactive feature, students can ask questions and participate in discussions at any time during the course, keeping close contact with the instructor and their classmates. This interactivity greatly facilitates student engagement and learning outcomes.

c. What is the classroom climate and student learning in a rain classroom setting?

In the teaching and learning environment of the rain classroom, the classroom atmosphere is usually energized and interactive. Students actively participate in the course through real-time interactions and discussions in the virtual space. They demonstrate a good learning state and show a high level of concentration and desire to learn.

Part VI: Other observations and recommendations

a. Do you think the Rain classroom model of teaching and learning is applicable to all subjects and programs?

The rainy day classroom model of teaching can be applied to most subjects and courses, especially those that can be learned through online interactions and discussions, such as mathematics and linguistics. However, for some practical subjects, it may be necessary to teach them in conjunction with hands-on practice.

b. What are your recommendations for nursing education based on Rainy Day?

On the "Rain Classroom" platform, real-time interaction and discussion functions can be fully utilized to provide a more interactive learning experience for nursing education. Students are encouraged to ask questions, share experiences, promote the exchange of ideas and deepen their understanding of nursing knowledge.

**Interview 2**

Part I: Personal Information

a. Age: I am 42 years old.

b. Education: I hold a Master's degree in Nursing.

c. Title: I am an associate professor.

d. Years of experience: I have been in nursing education for 18.

e. What you teach: health assessment

f. Duration and frequency of use: I have been using RainClass for two years and basically utilize it every week in my courses.

Part II: Usage of the Rain Classroom

1. What is your purpose for using Rain Classroom in nursing education?

Provides high-quality online nursing education resources, including video courses, textbooks, and practice guides.

b.What instructional activities have been conducted using the rainy day classroom in nursing education? (e.g., classroom lectures, group discussions, assignments, etc.)

Many teaching and learning activities are conducted in nursing education using the rain classroom, including classroom lectures, group discussions, hands-on demonstrations, and homework assignments.

What do you think are the advantages of using the rain classroom for teaching in nursing education?

I think there are many advantages of using rain classroom in nursing education. Firstly, it enables students to perform practical exercises in a virtual environment, which enhances their practical skills. Secondly, it provides real-time interactive features that make the classroom more active and lively. In addition, Rain Classroom can record students' performance, making it easy to assess and track learning progress at a later stage.

d.What challenges have you encountered in implementing blended learning supported by rainy day classroom nursing education? How were these challenges addressed?

One of the main challenges I encountered while implementing blended learning supported by Rain classroom Nursing Education was unstable internet connection. Students may be in different geographic locations and face issues with unstable internet connections, which affects the smooth flow of online learning.

The solution is to advise students to participate in a stable online environment, and to provide alternate ways of learning, such as providing audio recordings of lessons or text materials, so that students can learn even when the network is unstable.

Part III: Functions

a. What features do you use in your rain classroom instruction? How do these features help your teaching?

Features that I use frequently in Rain Classroom instruction include real-time interaction, where teachers can interact with students, answer questions, and provide guidance and feedback through Rain Classroom's real-time chat or discussion feature.

b. What is your favorite Rain Classroom feature? Why?

I especially like the simulated practice and case study features. With this feature, I can provide students with simulated practice scenarios of nursing skills and allow them to practice real-world skills in a virtual environment, which is crucial for nursing education.

Part IV: Teaching satisfaction

1. Are you satisfied with your teaching in the rain classroom? Why?

I am very happy with Rain Classroom Teaching. It has provided me with a wealth of tools and resources to make the classroom more lively and interesting.

1. Do you think rain classrooms improve student learning? Please provide examples.

The rain classroom has a significant impact on student learning outcomes. Through simulated practice and case studies, students were able to perform practical exercises in a virtual environment, which is crucial to the learning of the nursing profession. For example, we used the platform to simulate a first aid scenario, and students received effective training in practical skills and improved their ability to respond to emergencies.

1. What are the advantages and disadvantages of using the rain classroom as a teaching aid to implement blended learning in nursing education?

Students can interact and discuss in real time through the rain classroom, asking questions and sharing experiences, which enhances communication and interaction between students and teachers as well as their classmates. Compared with traditional teaching, rain classroom may lack face-to-face communication and interaction, and some practical nursing skills may not be fully demonstrated in the virtual environment.

d. Do you have any suggestions for improvements to the Rain Room?

Optimize the simulation practice experience. Add more simulation practice in real-life scenarios and provide more hands-on opportunities for nursing skills to help students better meet the challenges of real-life nursing.

Part V: Evaluation of Teaching Effectiveness

a. How would you characterize the performance and achievement of the students in the Rain Room?

The performance and achievement of students in the Rain Classroom have shown significant improvement. Through the diversified teaching resources and interactive functions provided by the platform, students participated more actively in the lessons. Many students achieved impressive results in quizzes and assignments, demonstrating their enthusiasm and hard work.

b. How do you think the rain classroom compares to traditional teaching methods in terms of student engagement and interaction?

Rain Classroom provides students with a more convenient, real-time interactive experience. Compared to traditional teaching methods, students can ask questions and share ideas on the online platform at any time, and interact with teachers and classmates in a more flexible and positive way.

c. What is the classroom climate and student learning in a rain classroom setting?

Rain Classroom creates a positive, interactive classroom atmosphere. Students actively participated in the course through online discussions and real-time interactions in this virtual environment. They demonstrated good learning status and showed great interest in the course content.

Part VI: Other observations and recommendations

a. Do you think the Rain classroom model of teaching and learning is applicable to all subjects and programs?

The rainy day classroom model of teaching and learning is applicable in many subjects and courses, especially in more theoretical subjects such as mathematics and literature. However, some practical and experimental subjects may require a combination of field practice and are not suitable for relying exclusively on online teaching.

b. What are your recommendations for nursing education based on Rainy Day?

Combined with the simulation and case analysis functions provided by "Rain Classroom", we design a rich variety of nursing scenarios, allowing students to perform actual operations in the virtual environment, practicing actual nursing skills and enhancing their practical abilities.

**Interview 3**

Part I: Personal Information

a. Age: I am 26 years old.

b. Education: I have a Master's degree.

c. Title: I am a lecturer.

d. Years of experience: I have been working in the field of nursing education for 4.

e. What you teach: Basic Nursing Science

f. Duration and frequency of use of RainClass: I have been using RainClass for two years and I will use it every week in my practicum sessions.

Part II: Usage of the Rain Classroom

a. What is the purpose of your use of the rain classroom in nursing education?

The purpose of using the rain classroom in nursing education is to provide students with the opportunity to interact with professional educators in order to address their questions and concerns during the learning process.

b.What instructional activities have been conducted using the rainy day classroom in nursing education? (e.g., classroom lectures, group discussions, assignments, etc.)

A variety of teaching and learning activities have been conducted in nursing education using Rain Classroom, including classroom lectures, group discussions, hands-on simulations, and online assignments.

c. What do you think are the advantages of using the rain classroom for teaching in nursing education?

I believe that there are many advantages to using Rain Classroom in nursing education. First, it provides a safe virtual environment in which students can practice operations, reducing the risk of hands-on courses. Secondly, it records every step of the student's maneuvers, which facilitates post-assessment and guidance.

d.What challenges have you encountered in implementing blended learning supported by rainy day classroom nursing education? How were these challenges addressed?

The challenge is course design and preparation of teaching resources. Blended learning in the rain classroom requires teachers to prepare online teaching resources and activities in advance, which may require additional time and effort.

SOLUTION: Plan lessons in advance and prepare instructional materials and resources to ensure that students can be guided smoothly through learning activities as the lesson progresses.

Part III: Functions

a. What features do you use in your rain classroom instruction? How do these features help your teaching?

I often use simulated practice and case studies in my rainy day classroom instruction, practicing various nursing skills in a virtual environment through simulated practice videos or interactive simulations

b. What is your favorite Rain Classroom feature? Why?

My favorite feature is the personalized guidance, the teaching teacher can push the operation video to the nursing students through the rain classroom, the nursing students watch the video in advance, ask questions, the teaching teacher to answer; choose a nursing student combined with the clinical case to operate on-site, the other nursing students to comment and correct the errors, point out the shortcomings in the process of the operation, the teaching teacher evaluation, and then the nursing students then perform a complete operation demonstration, the teaching teacher Then the nursing students will perform a complete operation demonstration again, and the instructor will explain each step and easy to deduct points in detail.

Part IV: Teaching satisfaction

a. Are you satisfied with your instruction in the rain classroom? Why?

I am very pleased with the use of Rain Classroom for instruction. Students can choose to participate in the program on their own schedule, providing greater flexibility.

b. Do you think the rain classroom has improved student learning? Please provide examples.

In my observations, the use of Rain Classroom has significantly improved student learning. Through online discussions and interactions, students were more actively engaged in the course and engaged in deeper conversations with the instructor and their peers. As an example, we had a discussion on chronic disease management in which students shared their practical experiences, facilitating the sharing and deepening of knowledge.

c. What are the advantages and disadvantages of using the rain classroom as a teaching aid to implement blended learning in nursing education?

The rain classroom offers simulated practice, which allows students to perform actual operations in a virtual environment, effectively enhancing their practical skills and preparing them for future field practice. The disadvantage is the high demand for self-discipline. Under the blended learning model, students need to possess a certain degree of self-discipline and independent learning ability, and be able to consciously organize their study time and manage their study progress.

d. Do you have any suggestions for improvements to the Rain Room?

The improvement is to provide more online resources. Expanding the library of teaching resources, including videos, cases, references, etc., enriches students' learning resources and helps them understand nursing more comprehensively.

Part V: Evaluation of Teaching Effectiveness

a. How would you characterize the performance and achievement of the students in the Rain Room?

1. We found that the Rain Classroom provides students with a quality learning experience. Through the rich teaching resources and real-time interactive features, students are able to learn and participate in discussions in a more flexible manner. In terms of course assessment, many students have achieved excellent results, reflecting their learning outcomes and the effective delivery of quality teaching.

b. How do you think the rain classroom compares to traditional teaching methods in terms of student engagement and interaction?

In the rain classroom, students can easily communicate with their teachers and classmates through real-time interaction and discussion features. Compared to traditional classroom teaching, this approach eliminates time and space constraints and allows students to participate more freely in the teaching process.

c. What is the classroom climate and student learning in a rainwater classroom setting?

In the teaching and learning environment of Rain Classroom, the classroom atmosphere seems to be very active and enthusiastic. Students fully expressed their views and thoughts through online discussions and real-time interactions. They maintained a good learning condition and showed a proactive learning attitude.

Part VI: Other observations and recommendations

a. Do you think the Rain classroom model of teaching and learning is applicable to all subjects and programs?

The Rain Classroom model of teaching and learning is applicable to most subjects and courses. In particular, theoretical subjects that can be learned through online discussions and interactions, such as social sciences and humanities, are well suited to be taught on this platform.

b. What are your recommendations for nursing education based on Rainy Day?

Through the "Rain Classroom" platform, online quizzes on nursing subjects can be released regularly to assess students' mastery of knowledge in a timely manner, provide a basis for teaching adjustments, and ensure the quality of teaching.

**Interview 4**

Part I: Personal Information

a. Age: I am 42 years old.

b. Education: I hold a Master's degree in Nursing.

c. Title: I am an associate professor.

d. Years of experience: I have been in nursing education for 18.

e. What you teach: I teach primarily internal medicine nursing, including nursing theory and practice.

f. Duration and frequency of use: I have been using RainClass for 3 years and basically use it every week in my courses.

Part II: Usage of the Rain Classroom

a. What is the purpose of your use of the rain classroom in nursing education?

The purpose of using the rain classroom in nursing education is to enhance classroom interaction and student engagement, as well as to better demonstrate actual nursing practice.

b.What instructional activities have been conducted using the rainy day classroom in nursing education? (e.g., classroom lectures, group discussions, assignments, etc.)

Many teaching and learning activities are conducted in nursing education using the rain classroom, including classroom lectures, group discussions, hands-on demonstrations, and homework assignments.

c. What do you think are the advantages of using the rain classroom for teaching in nursing education?

I think there are many advantages of using rain classroom in nursing education. Firstly, it enables students to perform practical exercises in a virtual environment, which enhances their practical skills. Secondly, it provides real-time interactive features that make the classroom more active and lively. In addition, Rain Classroom can record students' performance, making it easy to assess and track learning progress at a later stage.

d.What challenges have you encountered in implementing blended learning supported by rainy day classroom nursing education? How were these challenges addressed?

One of the main challenges I encountered while implementing blended learning supported by Rain classroom Nursing Education was keeping students engaged. In a virtual environment, students may face distraction and lack of engagement. The solution is to design rich and varied interactive activities that encourage students to participate in discussions and interactions, as well as provide regular and timely feedback and encouragement to maintain active student participation.

Part III: Functions

a. What features do you use in your rain classroom instruction? How do these features help your teaching?

A feature I use frequently in Rainclassroom teaching is homework posting and grading, where homework assignments are posted on Rainclassroom and students can complete and submit them via the platform within a specified time frame.

b. What is your favorite Rain Classroom feature? Why?

My favorite feature is the personalized guidance, the teaching teacher can push the operation video to the nursing students through the rain classroom, the nursing students watch the video in advance, ask questions, the teaching teacher to answer; choose a nursing student combined with the clinical case to operate on-site, the other nursing students to comment and correct the errors, point out the shortcomings in the process of the operation, the teaching teacher evaluation, and then the nursing students then perform a complete operation demonstration, the teaching teacher Then the nursing students will perform a complete operation demonstration again, and the instructor will explain each step and easy to deduct points in detail.

Part IV: Teaching satisfaction

a. Are you satisfied with your instruction in the rain classroom? Why?

I am very satisfied with Rain Classroom's teaching, which provides online interactive features that enable real-time communication and discussion between students and teachers to promote learning interactions.

b. Do you think the rain classroom has improved student learning? Please provide examples.

I think Rain Classroom has played a positive role in improving student learning. Through real-time interactions and discussions, students are more actively engaged in the classroom, asking questions and sharing ideas. For example, we conducted a simulation of perioperative care through the platform, and students got hands-on training in a virtual environment, which is difficult to achieve in a traditional classroom.

c. What are the advantages and disadvantages of using the rain classroom as a teaching aid to implement blended learning in nursing education?

The advantages of using Rain Classroom as a teaching aid to implement blended learning in nursing education are that it can enhance students' hands-on skills and engagement. The disadvantage may be that some technical training is required to ensure that all students are able to use the platform successfully.

d. Do you have any suggestions for improvements to the Rain Room?

For the improvement of the Rain Room, I suggest that the personalized guidance and feedback functions can be further enhanced to provide more targeted advice and tutoring based on students' learning performance and needs.

Part V: Evaluation of Teaching Effectiveness

a. How would you characterize the performance and achievement of the students in the Rain Room?

In the Rain Room, we noticed an overall positive trend in students' learning performance. They were active in course discussions and homework completion, demonstrating a good attitude to learning. In terms of grades, most students achieved grades that met or exceeded expectations, which demonstrated the effectiveness of their learning.

b. How do you think the rain classroom compares to traditional teaching methods in terms of student engagement and interaction?

Compared with traditional teaching methods, Rain Classroom enables students to ask and answer questions instantly during the course and stay in close contact with the teacher and their classmates through its real-time interactive features. This greatly promotes student engagement and learning compared to traditional teaching methods.

c. What is the classroom climate and student learning in a rainwater classroom setting?

The teaching and learning environment of a rain classroom usually creates an atmosphere of positive interaction. Students actively participate in the course through online discussions and real-time interactions. They remain in good learning mode and show a high level of concentration and enthusiasm for learning.

Part VI: Other observations and recommendations

a. Do you think the Rain classroom model of teaching and learning is applicable to all subjects and programs?

The rainy day classroom model of teaching can be applied to most subjects and courses, especially the more theoretical ones, such as mathematics and physics. However, some practical and experimental subjects may need to be taught in the field.

b. What are your recommendations for nursing education based on Rainy Day?

Utilizing the resource sharing function of "Rain Classroom" to share relevant reference materials and case studies of nursing discipline to enrich students' learning resources and help them to learn in depth.

**Interview 5**

Part I: Personal Information

a. Age: I am 38 years old.

b. Education: I hold a doctorate in nursing.

c. Title: I am an associate professor.

d. Years of experience: I have been in nursing education for 17 years.

e. What you teach: I primarily teach surgical nursing

f. Duration and frequency of use of RainClass: I have been using RainClass for 3 years and basically utilize it every week in my courses.

Part II: Usage of the Rain Classroom

a. What is the purpose of your use of the rain classroom in nursing education?

The purpose of using the rain classroom in nursing education is to provide simulated practice and case studies to help students apply theoretical knowledge to real-world nursing scenarios.

b.What instructional activities have been conducted using the rainy day classroom in nursing education? (e.g., classroom lectures, group discussions, assignments, etc.)

A variety of teaching and learning activities have been conducted in nursing education using Rain Classroom, including classroom lectures, group discussions, hands-on simulations, and online assignments.

c. What do you think are the advantages of using the rain classroom for teaching in nursing education?

I believe that there are many advantages to using Rain Classroom in nursing education. The interactive features of Rain Classroom allow students to communicate in real time with the instructor and classmates to discuss cases and solve problems, which promotes learning.

d.What challenges have you encountered in implementing blended learning supported by rainy day classroom nursing education? How were these challenges addressed?

One of the challenges I encountered when implementing blended learning supported by rainy day classroom nursing education was the difficulty in assessing students' hands-on skills. It is difficult to assess practical nursing skills in a virtual environment. The solution was to design targeted labs or simulated practice activities in conjunction with field practice to ensure that students had the opportunity to practice and apply nursing skills in a real-world setting.

Part III: Functions

a. What features do you use in your rain classroom instruction? How do these features help your teaching?

I often use online quizzes and assessments in my Rain Classroom instruction, utilizing Rain Classroom to administer online quizzes and exams to assess student learning outcomes in a timely manner and help them consolidate their knowledge and skills

b. What is your favorite Rain Classroom feature? Why?

I especially like the homework posting and grading feature. It allows me to provide students with practice questions so they can practice and reinforce what they have learned at home. Also, I can correct assignments on the platform and give specific advice and feedback.

Part IV: Teaching satisfaction

a. Are you satisfied with your instruction in the rain classroom? Why?

I am very happy with using Rain Classroom for teaching. It adds to my lessons and makes teaching come alive.

b. Do you think the rain classroom has improved student learning? Please provide examples.

Rain Classroom has played an active role in improving student learning outcomes. Through online quizzes and assessments, we are able to keep track of student learning and provide targeted feedback and guidance. For example, we conducted regular quizzes on nursing knowledge, and students were able to review and improve with timely feedback, which is important for knowledge consolidation.

c. What are the advantages and disadvantages of using the rain classroom as a teaching aid to implement blended learning in nursing education?

Teachers can share rich teaching resources in the rain classroom, including courseware, reference materials, etc., which provides students with more learning resources and enriches the teaching content. The disadvantage may be that teachers need to have the ability to design online courses and guide students in independent learning, and the teaching design requirements are relatively high.

1. Do you have any suggestions for improvements to the Rain Room?

I suggest that Rain Classroom can enhance the data analysis and assessment functions. Provide more comprehensive learning data analysis reports so that teachers can clearly understand the learning situation of students in order to adjust teaching strategies in a timely manner.

Part V: Evaluation of Teaching Effectiveness

a. How would you characterize the performance and achievement of the students in the Rain Room?

Students' academic performance and achievement in the Rain Room is outstanding. They demonstrate strong motivation to learn by actively participating in discussions and completing assignments. In terms of grades, many students performed well on quizzes and assignments, reflecting the seriousness and effort they put into the course.

b. How do you think the rain classroom compares to traditional teaching methods in terms of student engagement and interaction?

Rain Classroom provides a more convenient and active learning environment for students through real-time interaction. Compared to traditional teaching methods, students can ask questions and participate in discussions more freely, and interact more closely and efficiently with their teachers and classmates.

c. What is the classroom climate and student learning in a rain classroom setting?

In a rain classroom, the classroom atmosphere is usually very active. Students actively participate in the course through real-time interactions and discussions. They stay in good shape and show a positive attitude towards learning.

Part VI: Other observations and recommendations

a. Do you think the Rain classroom model of teaching and learning is applicable to all subjects and programs?

The Rain Classroom model of teaching and learning can be used effectively in many subjects and programs. In particular, subjects that can be learned through online interactions and real-time discussions, such as sociology and psychology, are well suited to be taught on this platform....

b. What are your recommendations for nursing education based on Rainy Day?

In the "rain classroom", group discussions and team projects can be set up to allow students to work together to solve practical nursing problems and develop teamwork and problem-solving skills.

**Interview 6**

Part I: Personal Information

a. Age: I am 50 years old.

b. Education: I hold a doctorate in nursing.

c. Title: I am a professor.

d. Years of experience: I have been in nursing education for 23 years.

e. What you teach: I primarily teach Introduction to Nursing

f. Duration and frequency of use of RainClass: I have been using RainClass for 3 years and basically utilize it every week in my courses.

Part II: Usage of the Rain Classroom

a. What is the purpose of your use of the rain classroom in nursing education?

The purpose of using Rain Classroom in nursing education is to provide online quizzes and assessments to help students test their learning outcomes and understanding.

b.What instructional activities have been conducted using the rainy day classroom in nursing education? (e.g., classroom lectures, group discussions, assignments, etc.)

A variety of teaching and learning activities have been conducted in nursing education using Rain Classroom, including classroom lectures, group discussions, hands-on simulations, and online assignments.

c. What do you think are the advantages of using the rain classroom for teaching in nursing education?

I believe that there are many advantages to using Rain Classroom in nursing education. First, it provides a safe virtual environment in which students can practice operations, reducing the risk of hands-on courses. Secondly, it records every step of the students' maneuvers, which facilitates post-assessment and guidance. By sharing real or fictional nursing cases, students can have in-depth discussions and analysis in the rain classroom to deepen their understanding of practical work.

d.What challenges have you encountered in implementing blended learning supported by rainy day classroom nursing education? How were these challenges addressed?

Balance of individualized instruction. Each student may have different learning needs and levels, and it is a challenge to personalize instruction in a virtual environment. The solution is to encourage students to learn independently according to their own learning needs by providing personalized guidance and support, while at the same time communicating with them regularly to understand their learning progress and difficulties and provide targeted assistance.

Part III: Functions

a. What features do you use in your rain classroom instruction? How do these features help your teaching?

In the rain classroom, I regularly use course assessment and feedback, personalized instruction The online quiz function conducts regular knowledge tests to assess students' mastery of nursing subject knowledge and provide timely feedback; personalized guidance and advice are provided based on students' learning performance and needs to help them better understand and apply their nursing knowledge.

b. What is your favorite Rain Classroom feature? Why?

My favorite Rain Classroom feature is the resource sharing feature. In Rain Classroom, I can share rich teaching resources, such as course syllabus, reference materials, video links, etc., to help students learn deeply.

Part IV: Teaching satisfaction

a. Are you satisfied with your instruction in the rain classroom? Why?

I am very satisfied with the Rain Classroom instruction. This is because the use of the Rain Classroom hands-on training feature has improved the clinical practice of nursing students.

b. Do you think the rain classroom has improved student learning? Please provide examples.

In the nursing courses I have taught, I believe that the rain classroom has significantly improved student learning. Through real-time interaction and discussion, students were more actively engaged in the course and resolved many academic concerns. For example, we had a case discussion on cardiac emergencies, and students learned many practical nursing skills during the interaction, which would have been difficult to do in a traditional classroom.

c. What are the advantages and disadvantages of using the rain classroom as a teaching aid to implement blended learning in nursing education?

The advantage of using rain classroom as a teaching aid to implement blended learning in nursing education is that students can learn freely according to their own schedule, without the limitation of location and time, which is conducive to enhancing the flexibility and autonomy of learning. The disadvantage is the dependence on equipment, students need to have the appropriate equipment to carry out online learning, for some resource-poor areas or students there may be some difficulties.

d. Do you have any suggestions for improvements to the Rain Room?

I suggest that Rain Classroom could add enhanced mobile support. Optimize the mobile user experience to ensure that students can easily learn and interact on their phones or tablets.

Part V: Evaluation of Teaching Effectiveness

a. How would you characterize the performance and achievement of the students in the Rain Room?

We found that the Rain Room provided a good learning platform for students. Students have demonstrated a positive attitude towards learning in the course, and have enhanced their academic standards by participating in discussions and completing assignments. In terms of assessment, many students have achieved encouraging results, demonstrating a good combination of teaching quality and student learning outcomes.

b. How do you think the rain classroom compares to traditional teaching methods in terms of student engagement and interaction?

Compared to traditional teaching methods, rain classrooms are more likely to stimulate student participation and interaction, making the classroom more active.

c. What is the classroom climate and student learning in a rain classroom setting?

The classroom atmosphere in a rainy day classroom setting is often very lively and positive. Students participate enthusiastically in the course through real-time interactions and discussions. They remain in good learning mode and demonstrate a thirst and love for knowledge.

Part VI: Other observations and recommendations

a. Do you think the Rain classroom model of teaching and learning is applicable to all subjects and programs?

The rain classroom model is applicable to many subjects and courses, especially those theoretical subjects that can be learned through online interaction and real-time discussions, such as history and literature, which are well suited to be taught on this platform.

b. What are your recommendations for nursing education based on Rainy Day?

Based on the rain classroom, I suggest that more sharing and discussion of real-life cases could be included in nursing education, as well as

Consideration should be given to adding some virtual laboratory functions to enhance students' practical skills. Using the personalized guidance function of "Rain Classroom", personalized teaching suggestions and guidance are provided to students according to their learning performance and needs, helping them to fully understand and apply their nursing knowledge.

**Interview 7**

Part I: Personal Information

a. Age: I am 45 years old.

b. Education: I hold a Master's degree in Nursing.

c. Title: I currently hold the position of Associate Professor.

d. Years of experience: I have worked in the field of nursing education for 19.

e. What I teach: I mainly teach obstetrics and gynecology nursing

f. Duration and frequency of use of RainClass: I have been using RainClass for 3 years and basically utilize it every week in my courses.

Part II: Usage of the Rain Classroom

a. What is the purpose of your use of the rain classroom in nursing education?

The purpose of using the rain classroom in nursing education is to provide a flexible learning environment that promotes a personalized learning experience for students with varying learning needs and paces.

b.What instructional activities have been conducted using the rainy day classroom in nursing education? (e.g., classroom lectures, group discussions, assignments, etc.)

A variety of teaching and learning activities have been conducted in nursing education using Rain Classroom, including classroom lectures, group discussions, hands-on simulations, and online assignments.

c. What do you think are the advantages of using the rain classroom for teaching in nursing education?

I believe that there are many advantages to using rain classrooms in nursing education. Students can study with peers from different regions or even different countries, thus expanding their horizons and learning about nursing practice experiences in different regions.

d.What challenges have you encountered in implementing blended learning supported by rainy day classroom nursing education? How were these challenges addressed?

The challenge I encountered when implementing blended learning supported by Rainclassroom Nursing Education was timely communication and feedback. In a virtual environment where communication between faculty and students may be somewhat limited, it is a challenge to maintain timely communication and feedback. Effective communication between faculty and students is maintained by establishing clear lines of communication, encouraging students to ask questions and make comments, and responding to student questions and feedback in a timely manner.

Part III: Functions

a. What features do you use in your rain classroom instruction? How do these features help your teaching?

In the rain classroom teaching, I often use operation training Through the rain classroom operation video pushed to the nursing students, nursing students watch the video in advance, put forward questions, teaching teachers to answer; choose a nursing student combined with the clinical case to operate on-site, other nursing students to comment and correct errors, point out the shortcomings of the operation process, teaching teachers to evaluate, and then the nursing students then carry out a complete demonstration of the operation, teaching teachers to explain in detail the various steps and easy to deduction points.

b. What is your favorite Rain Classroom feature? Why?

I highly value the course evaluation and feedback functions. Through the platform's evaluation function, I can understand students' satisfaction and understanding of the course, so that I can make corresponding adjustments to the teaching methods and contents to improve the quality of teaching.

Part IV: Teaching satisfaction

a. Are you satisfied with your instruction in the rain classroom? Why?

Overall satisfied and very convenient to use for situations where face-to-face instruction is not possible.

b. Do you think the rain classroom has improved student learning? Please provide examples.

I have observed that Rain Classroom has had a positive impact on student learning outcomes. Through real-time interactions and discussions, students were more actively engaged in the classroom and asked many valuable questions. For example, we started a discussion on pain assessment and management through the platform, and the students gained an interactive insight into the principles and methods of pain management, which will be very helpful for their future practice.

c. What are the advantages and disadvantages of using the rain classroom as a teaching aid to implement blended learning in nursing education?

The advantages of implementing blended learning in nursing education using the rain classroom as a teaching aid are the ability to enhance practice, the disadvantage may be that some technical training is required to ensure that all students are able to use it successfully.

d. Do you have any suggestions for improvements to the Rain Room?

Provide a platform for sharing more examples of practice. Create a platform where students and teachers can share successes and lessons learned in practice and promote the integration of practice and theory.

Part V: Evaluation of Teaching Effectiveness

a. How would you characterize the performance and achievement of the students in the Rain Room?

The performance and achievement of students in the Rain Room is encouraging. They actively participated in the course by participating in discussions and completing assignments, showing a high level of motivation. In terms of grades, the majority of students achieved excellent grades, reflecting their serious commitment and hard work in the course.

b. How do you think the rain classroom compares to traditional teaching methods in terms of student engagement and interaction?

In the rain classroom, students can communicate with teachers and classmates anytime, anywhere through the real-time interactive function. This provides students with a larger space for participation and a more convenient interactive experience than traditional teaching methods, which promotes the improvement of learning results.

c. What is the classroom climate and student learning in a rainwater classroom setting?

In a rain classroom, the classroom atmosphere often seems very lively and energetic. Students are actively engaged in the course through real-time interactions and discussions. They maintain a good learning state and show a strong desire for knowledge.

Part VI: Other observations and recommendations

a. Do you think the Rain classroom model of teaching and learning is applicable to all subjects and programs?

The rainy day classroom model of teaching and learning can be applied to most subjects and courses, especially those that can be learned through online interaction and discussion, such as computer science and social science. However, some of the more practical subjects may require a combination of field practice and are not suitable for relying entirely on online teaching.

b. What are your recommendations for nursing education based on Rainy Day?

It is recommended to collect students' feedback on the "Rain Classroom" teaching mode on a regular basis to understand their learning experience and needs, so as to optimize the teaching methods and contents in a timely manner and enhance the teaching effect.

**Interview 8**

Part I: Personal Information

a. Age: I am 55 years old.

b. Education: I hold a doctorate in nursing.

c. Title: I am currently a professor.

d. Years of experience: I have been working in the field of nursing for 28 years.

e. What I teach: Pediatric Nursing.

f. Length of time and frequency of use of RainClass: I have been using RainClass for three years, about once a week.

Part II: Usage of the Rain Classroom

a. What is the purpose of your use of the rain classroom in nursing education?

The purpose of using the rain classroom in nursing education is to provide education through an online platform that offers students who are unable to attend a traditional classroom setting a way to obtain a quality nursing education.

b.What instructional activities have been conducted using the rainy day classroom in nursing education? (e.g., classroom lectures, group discussions, assignments, etc.)

A variety of teaching and learning activities were conducted in nursing education using Rain Classroom, including classroom lectures, group discussions, hands-on simulations, homework assignments, online quizzes, and exams.

c. What do you think are the advantages of using the rain classroom for teaching in nursing education?

Advantage Moment Online Assessment and Exam, teachers can utilize Rain Classroom to conduct online quizzes and exams to assess students' learning outcomes in a timely manner and help them consolidate their knowledge and skills.

d.What challenges have you encountered in implementing blended learning supported by rainy day classroom nursing education? How were these challenges addressed?

Guaranteeing the quality and effectiveness of teaching and learning. Under the blended learning model, how to guarantee the quality and effectiveness of teaching is an important challenge. Teaching quality can be improved by conducting regular teaching assessment and feedback, collecting students' opinions and suggestions, and making corresponding adjustments and optimization according to the feedback results.

Part III: Functions

a. What features do you use in your rain classroom instruction? How do these features help your teaching?

In the rain classroom teaching, I often use the pre-study effect test through the rain classroom to send individual test questions to the nursing students, the lead teacher through the background to understand the knowledge mastery and weak points of the nursing students.

b. What is your favorite Rain Classroom feature? Why?

I especially appreciate the real-time interaction and discussion features. This allows me to communicate with students in real time, answer questions, facilitate the exchange of ideas, and make the course more interactive and engaging.

Part IV: Teaching satisfaction

a. Are you satisfied with your instruction in the rain classroom? Why?

I am very happy with using Rain Classroom for my teaching.

b. Do you think the rain classroom has improved student learning? Please provide examples.

I believe that Rain Classroom has made significant improvements to nursing education. Through real-time interactions and discussions, students were more actively engaged in the program and solved many challenges in nursing practice. For example, we used the platform to conduct a case study on diabetes care, and students were able to improve their understanding and application of diabetes care through hands-on practice and discussion.

c. What are the advantages and disadvantages of using the rain classroom as a teaching aid to implement blended learning in nursing education?

The advantages of implementing blended learning in nursing education using the rain classroom as a teaching aid are the ability to enhance practice, the disadvantage may be that some technical training is required to ensure that all students are able to use it successfully.

d. Do you have any suggestions for improvements to the Rain Room?

I would suggest that Rain Classroom could enhance faculty training and support. Provide teachers with more training resources on online teaching and instructional design to help them better utilize the Rain Classroom for nursing education.

Part V: Evaluation of Teaching Effectiveness

a. How would you characterize the performance and achievement of the students in the Rain Room?

We can clearly see that the Rain Classroom has had a positive impact on student performance and achievement. Students are actively engaged in learning through online discussions and real-time interactions, demonstrating good academic performance. In course assessments, many students have achieved significant improvements in their grades, demonstrating the platform's effective support for teaching quality.

b. How do you think the rain classroom compares to traditional teaching methods in terms of student engagement and interaction?

Rain Classroom allows students to participate more flexibly in classroom discussions through the function of real-time interaction. Compared to traditional teaching methods, this interactive format allows students to ask questions and share ideas more freely, interacting more closely with the teacher and classmates, and helping to improve learning outcomes.

c. What is the classroom climate and student learning in a rainwater classroom setting?

In a rain classroom teaching environment, the classroom atmosphere usually seems very active and interactive. Students are actively engaged in the course through real-time interactions and discussions. They maintain a good learning state and show a high level of commitment to learning. Part VI: Other Observations and Recommendations

a. Do you think the Rain classroom model of teaching and learning is applicable to all subjects and programs?

The rainy day classroom model is applicable in many subjects and courses, especially those theoretical subjects that can be learned through online interactions and discussions, such as philosophy and political science, which are well suited to be taught on this platform.

b. What are your recommendations for nursing education based on Rainy Day?

Although online teaching methods are flexible and colorful, offline traditional teaching methods are more authoritative, direct, and more conducive to face-to-face emotional communication between teachers and students.
